# Design, synthesis, and anticancer activity of novel isocryptolepine ‘aza’ type acyl thiourea derivatives via combined experimental and computational approach

**DOI:** 10.1042/BSR20253519

**Published:** 2026-01-08

**Authors:** Ammara Fayyaz, Syeda Abida Ejaz, Atteeque Ahmed, Pervaiz Ali Channar, Saira Afzal, Rabail Ujan, Aamer Saeed, Rifhat Bibi, Bshra A. Alsfouk, Qamar Abbas, Reshma Sahito, Michael Bolte, Tuncer Hökelek

**Affiliations:** 1 Department of Pharmaceutical Chemistry, Faculty of Pharmacy, The Islamia University of Bahawalpur, 63100, Pakistan; 2 Sulaiman Bin Abdullah Aba Al-Khail-Centre for Interdisciplinary Research in Basic Sciences (SA-CIRBS), International Islamic University, Islamabad, 44000, Pakistan; 3 Department of Basic Science and Humanities, Faculty of Basic and Allied Sciences, Dawood University of Engineering and Technology, Karachi, 74800, Pakistan; 4 Faculty of Pharmacy, The University of Lahore, Lahore, Pakistan; 5 Department of MRC Liaquat, University of Medical & Health Sciences Jamshoro, Pakistan; 6 Department of Chemistry, Quaid-I-Azam University, Islamabad, 45320, Pakistan; 7 Fatima Jinnah Women University Rawalpindi, Rawalpindi, 46000, Pakistan; 8 Department of Pharmaceutical Sciences, College of Pharmacy, Princess Nourah bint Abdulrahman University, Riyadh, Saudi Arabia; 9 Department of Biology, College of Science, University of Bahrain, Sakhir, 32038, Bahrain; 10 Department of Zoology University of Sindh, Jamshoro, Pakistan; 11 Department Chemie, Institut fuer Anorganische ChemieJ.-W.-Goethe-UniversitaetMax-von-Laue-Str. 7D-60438 Frankfurt/Main, Germany; 12 Department of Physics, Faculty of Engineering, Hacettepe University, Beytepe-Ankara, Ankara, 06800, Turkey

**Keywords:** acyl thiourea, hyperpigmentation, kinetic studies, molecular modeling, structure–activity relationship, tyrosinase inhibitors, isocryptolepine

## Abstract

To identify novel tyrosinase inhibitors, a series of isocryptolepine ‘aza’ type acyl thiourea analogs (6a–6h) were designed and synthesized using a multistep strategy. Spectroscopic methods including FTIR, UV–vis, ^1^H NMR, ^13^C NMR, and EI-MS were utilized for detailed analysis of compounds. Their tyrosinase inhibitory activities were evaluated in vitro, demonstrating superior potency compared with kojic acid (IC_50_ = 16.83 ± 1.162 μM). The synthesized compounds exhibited IC_50_ values ranging from 0.832 ± 0.03 to 7.945 ± 0.63 μM, with compound **6g** emerging as the most potent inhibitor (IC_50_ = 0.832 ± 0.03 μM). Kinetic studies revealed competitive inhibition by compound **6g**, highlighting its potential as a lead candidate for treating tyrosinase-mediated hyperpigmentation. Additional evaluations showed that these compounds also effectively inhibited other enzymes involved in cancer progression, indicating their broad therapeutic potential. Molecular modeling studies against the tyrosinase enzyme (PDB: 4OUA) confirmed strong binding interactions, while structure-activity relationship analyses provided insights into their inhibitory mechanisms. Geometry optimization of the compounds, supporting their favorable molecular properties. Drug-likeness evaluations further validated the potential of these analogs as promising anti-tyrosinase agents. Overall, this study establishes compound **6g** and its analogs as compelling candidates for further development in hyperpigmentation and cancer therapeutics.

## Introduction

Skin cancer is a condition marked by an accumulation of malignant cells in the epidermis. It constitutes one of the most frequent types of cancer globally, accounting for approximately 3.3 million cases per year [1,2]. The increase in incidence has been ascribed to excessive skin exposure to solar or ultraviolet radiation (UV), resulting from the depletion of atmospheric ozone [1,3]. Melanoma is a severe manifestation of skin cancer characterized by an active proliferation of melanin-producing cells called melanocytes. Melanin plays an essential role in skin homeostasis by guarding against UV radiation and scavenging harmful chemical and drugs [4]. However, melanoma-induced melanin elevation leads to skin pigmentation and discoloration, as well as tumor formation [1]. Although surgical removal of the tumor is regarded as one of the most promising ways to treat skin cancer, it might cause disfigurement, necessitating more expensive skin grafts to conceal the resulting flaws. Therefore, targeting enzymes involved in tumor development is being considered as an alternative possibility [5].

Tyrosinase, also known as polyphenol oxidase, is a metalloprotein that is actively involved in the melanin production (melanogenesis) [6,7]. It acts as a rate-limiting enzyme for the first two reactions of melanin biosynthesis. In melanocytes, it hydroxylates L-tyrosine to 3,4-dihydroxyphenylalanine (L-DOPA) and oxidizes it to DOPA-quinone, which naturally polymerizes to form melanin [8]. . However, elevated levels of tyrosinase considerably contribute to the increased synthesis and accumulation of melanin in melanocytes [1]. While melanin primarily serves a photoprotective role in human skin, the excessive accumulation of melanin in certain localized areas leads to various skin diseases. Considering the role of tyrosinase in melanogenesis, it has been identified as a potential target to treat various pathological conditions associated with increased melanin level such as melanoma and pigmentary disorders [9]. Previously, a novel series of N-1 and C-3 substituted indole-based thiosemicarbazones has been reported against tyrosinase (IC₅₀ range from 12.4 to 47.2 μM) as melanoma inhibitors, with well-defined structure-activity relationship [10]. Hence, the researchers are in search of identification and characterization of tyrosinase inhibitors with less or no side effects.

In addition to tyrosinase, another enzyme, i.e. carbonic anhydrase (CAs), comprises a family of zinc metalloenzymes that catalyze the reversible hydration of CO_2_, in the presence of water, to bicarbonate with the release of a proton [11]. Recent studies have shown that its expression is induced in the endothelium of neo-vessels in melanoma and esophageal, renal, and lung cancer [12]. Novel heterocyclic scaffolds for CA inhibition have been reported in recent research [13], offering structural and mechanistic insights that can guide the development of CA-targeted drugs in tumor angiogenesis.

The third targeted enzyme, i.e., alkaline phosphatase, comprises a collection of diverse enzymes that facilitate the breakdown of monophosphate esters at alkaline pH. It has been identified in a variety of species, spanning from bacteria to humans [14]. A 2023 overview of N-, O-, and S-heterocycles as AP inhibitors showed numerous chemical moieties that successfully inhibited the AP activity [15]. On the basis of the above facts, the studies guided the scaffold selection for targeting alkaline phosphatase in metastatic melanoma.

The serum alkaline phosphatase of growing children is high because it is being formed in association with some active bone being produced and designed for development. Similarly, serum alkaline phosphatase is higher in males than females, but the difference diminishes after age 60. By contrast, females have a greater alkaline phosphatase in serum during adolescence, pregnancy, lactation, and especially menopause for physiological reasons [16]. However, high serum alkaline phosphatase levels due to a pathogenic cause must not be overlooked owing to the fact that the abnormal growth of neoplasms or tumors present in diverse body locations can cause over-production of alkaline phosphatase, which leaks into circulation. In addition, the presence of placental alkaline phosphatase has been shown to correlate with metastatic melanoma [17,18].

Recently, multi-target enzyme inhibition has garnered a lot of interest for the treatment of pigment-associated diseases including melanoma. For instance, a number of heterocyclic scaffolds have been shown to exhibit activity against carbonic anhydrases and to function as strong tyrosinase inhibitors, thus connecting the suppression of tumor-associated angiogenesis with the prevention of melanogenesis [19]. A recent study on hydroxypyridinone derivatives demonstrated their dual antioxidant and anti-tyrosinase potential, indicating that these scaffolds may be further designed for multi-enzyme variation in melanoma therapy [20]. Despite these advancements, there are limited reports on compounds that concurrently target tyrosinase, alkaline phosphatase, and carbonic anhydrase, highlighting the innovative potential of developing thiourea-based scaffolds that can address multiple melanoma-related pathways within a single chemotype.

**Figure 1:**
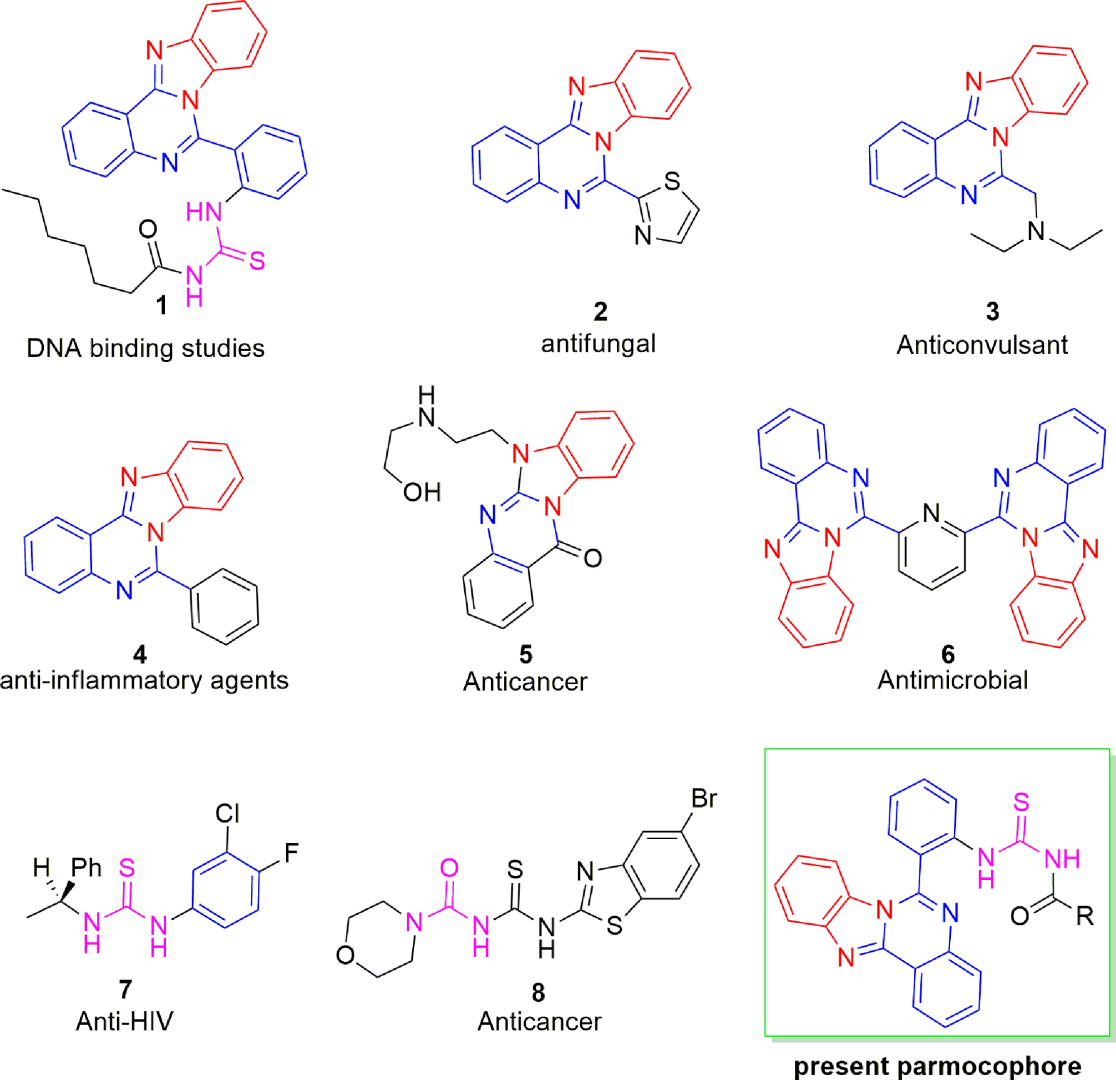
Isocryptolepine ‘aza’ type compounds and acyl thiourea structures [22–24].

Thiourea has been recognized as a versatile and pharmaceutically appropriate motif and has gained considerable attention due to their utility in synthesis and drug development. Recent studies on 3-hydroxypyridin-4-one derivatives containing benzyl hydrazide moieties have revealed their antioxidant and anti-tyrosinase activities, with IC₅₀ values ranging from approximately 25 to 64 μM. The most effective compound, 6i, exhibited an IC₅₀ of around 25 μM and was demonstrated through docking studies to bind stably within the active site of tyrosinase, acting as a competitive inhibitor and providing insights into the mechanisms of enzyme interaction [21]. . In addition, our group has significantly contributed towards the expansion of medicinally relevant chemical space while exploring new libraries of thiourea derivatives bearing structurally unique and balanced molecular complexity. These compounds have been tested against different therapeutic targets and remarkably produced promising leads for future drug (Figure 1) [22–24].

## Results and discussion

### Chemistry

#### Procedure for the synthesis of thiourea compounds

A small series of isocryptolepine ‘aza’ type acyl thioureas was synthesized by reacting benzene-1,2-diamine **2** with anthranilic acid in polyphosphoric acid at 150°C for 5 h in first. In the second step, amine **3** was reacted with freshly prepared isothiocyanate of different alkylated acid chlorides in dry acetone overnight at room temperature to obtain the final product. The crude product was recrystallized in ethanol to afford pure product.

The ^1^H NMR spectra displayed characteristic N-H protons as distinct singlets within the ranges of 12.64–13.29 ppm and 11.31–9.48 ppm for the N-H protons of both thioureas. The signals exhibited deshielding as a result of weak intermolecular interactions and intramolecular hydrogen bonding, suggesting the presence of a C = S bond adjacent to an N-H connection. Aromatic protons from various positions in all derivatives generated multiple signals between 6–9 ppm, while signals in the 0.5–2.4 ppm range indicated the presence of the alkyl component in the molecules. In the 13C NMR spectrum, signals at 180.09–179.5 ppm and 175.9–175.4 ppm are attributed to thiocarbonyl C = S and carbonyl C = O groups, respectively. Aromatic carbon signals were observed within the range of 113–148 ppm. The signals in the range of 13–35 ppm indicate the aliphatic alkyl groups.

#### Procedure

**Figure 2:**
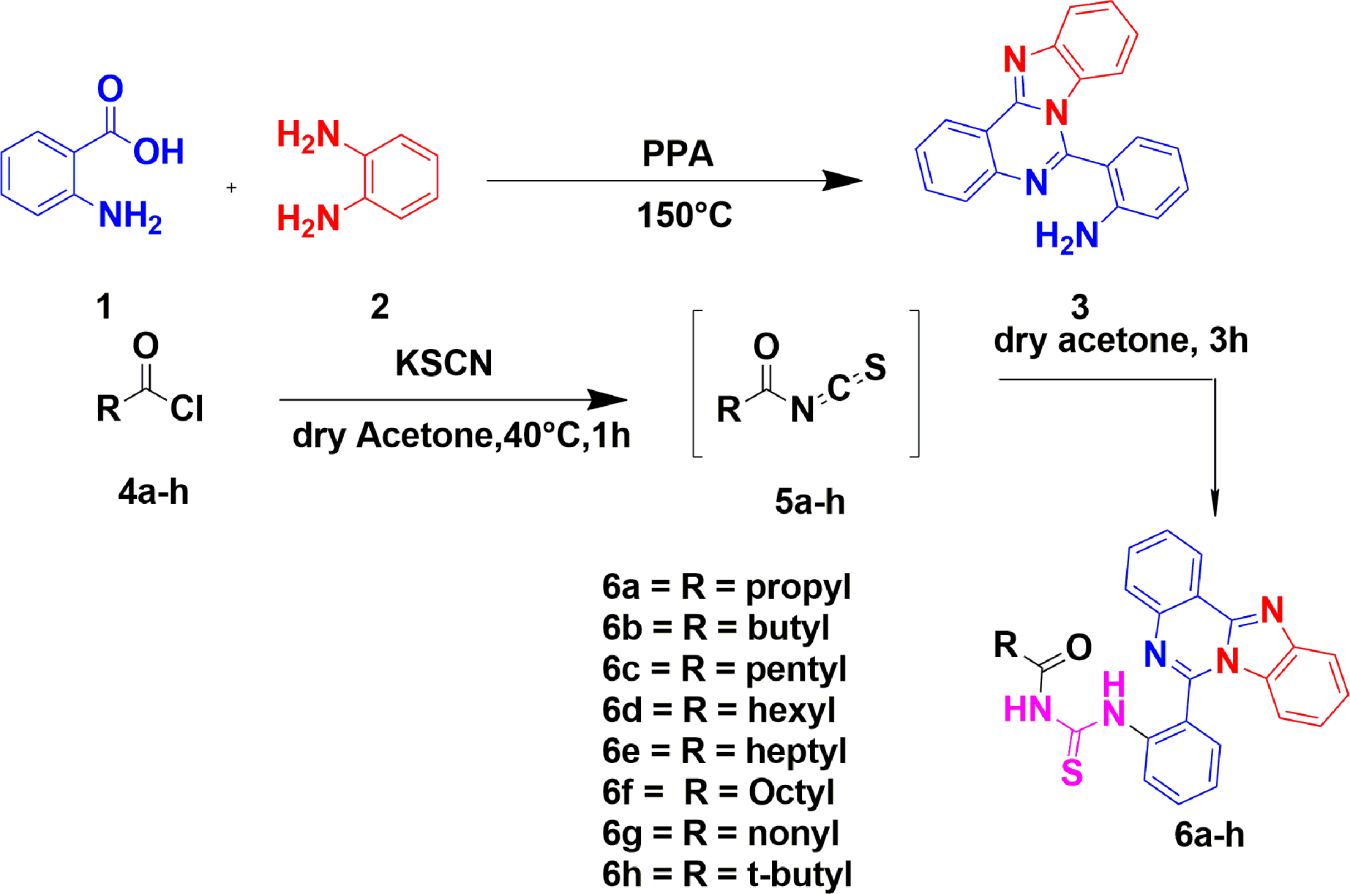
Synthetic route for isocryptolepine ‘aza’ type compounds acyl thioureas.

A two-neck round-bottom flask, fitted with a magnetic stirrer, was loaded with anthranilic acid (1) at 18.49 mmol (2.54 g), benzene-1,2-diamine (2) at 9.25 mmol (1.00 g), and PPA (8 ml). The reaction mixture was mixed at a temperature of 150°C for a duration of 5 h. Upon reaching room temperature, the resultant mixture was subjected to dilution with water and subsequently neutralized using a saturated solution of potassium carbonate (Figure 2). The product was precipitated, filtered, washed, and recrystallized using methanol to yield the pure desired intermediate **3**. The amine **3** was further converted to different alkyl-substituted acyl thiourea (**6**a–**h**) according to reported procedure [22–24].

#### X-ray crystallography

**Figure 3:**
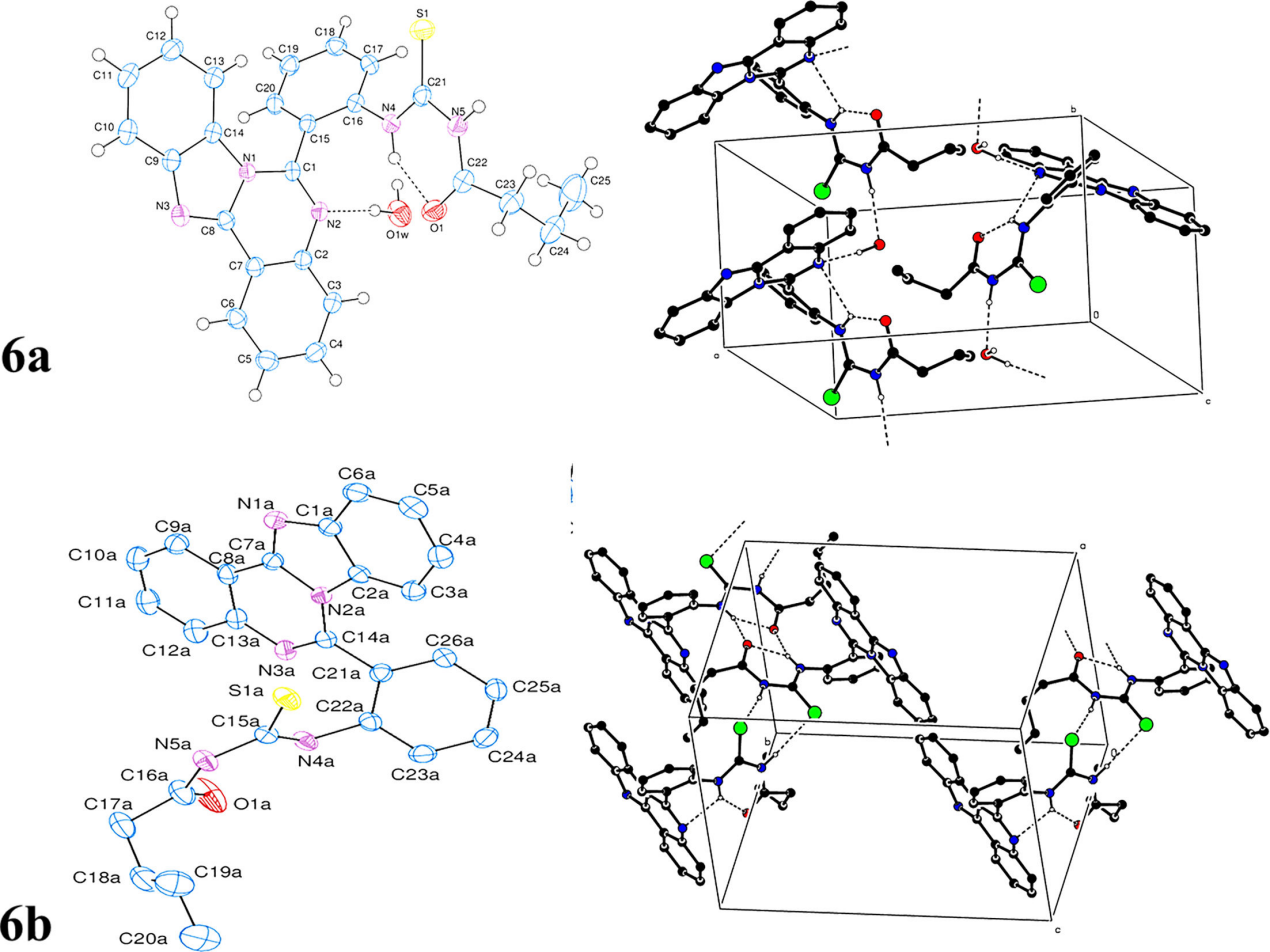
Thermal ellipsoids of 6a drawn at the 50% probability level (left side). A view along the a-axis of the crystal packing of the title compound. The N—H_Amn_ ∙∙∙ O_Bnz_, O—H_Hydr_ ∙∙∙ N_Amn_, N—H_Amn_ ∙∙∙ O_Hydr_ and C—H_Bnz_ ∙∙∙ O_Bnz_ hydrogen bonds (Amn = amino, Bnz = benzoate and Hydr = hydroxy) Hydrogen bonds are represented as dashed lines (refer to Table 1 ), and only the hydrogen atoms participating in these interactions are incorporated for clarity (right side).

The single crystal X-ray structural determination of the title compound confirms the structural assignment obtained from spectroscopic data. Table 1 provides a summary of the experimental details, encompassing crystal data, data collection, and refinement processes. Tables 1 and 2 present the hydrogen-bond geometry, selected interatomic distances, bond lengths, bond angles, and torsion angles, respectively. Figure 3 illustrates the molecular structure and the atom-numbering scheme.

**Table 1:** Hydrogen-bond geometry (Å,^o^) for compounds 6a, 6b

6a	D-H∙∙∙A	D-H	H∙∙∙A	D∙∙∙A	D-H∙∙∙A
	N4—H4···O1 N5—H5···O1Wiii O1W—H1WA···N2 C24—H24A···Cg3ii	0.90 (3) 0.87 (3) 0.96 (4) 0.99	1.89 (3) 2.00 (3) 1.94 (4) 2.98	2.645 (2) 2.872 (3) 3.888 (2) 3.784 (3)	140 (2) 174 (2) 173 (3) 142
**6b**	**D-H∙∙∙A**	**D-H**	**H∙∙∙A**	**D∙∙∙A**	**D-H∙∙∙A**
	N4—H4···O1 N4—H4···N3 N5—H5···S1Ai N4A—H4A···O1A N4A—H4A···O1Aii N5A—H5A···S1iii C17—H17A···Cg9iv	0.90 (2) 0.90 (2) 0.87 (2) 0.82 (2) 0.82 (2) 0.91 (2) 0.99	1.88 (2) 2.32 (2) 2.66 (2) 1.98 (2) 2.29 (2) 2.40 (2) 2.84	2.634 (2) 2.931 (2) 3.5161 (17) 2.653 (2) 2.971 (2) 3.3010 (17) 3.696 (2)	7 (19) 124.2 (17) 169.3 (19) 139 (2) 140 (2) 171.5 (19) 147

Symmetry codes: (i) *x*, *y*−1, *z*; (ii) −*x* + 1, −*y* + 2, −*z*; (iii) *x*, *y* + 1, *z*; (iv) 2−*x*, −*y*, 1−*z*.

#### Crystal structure of 6a

**Figure 4:**
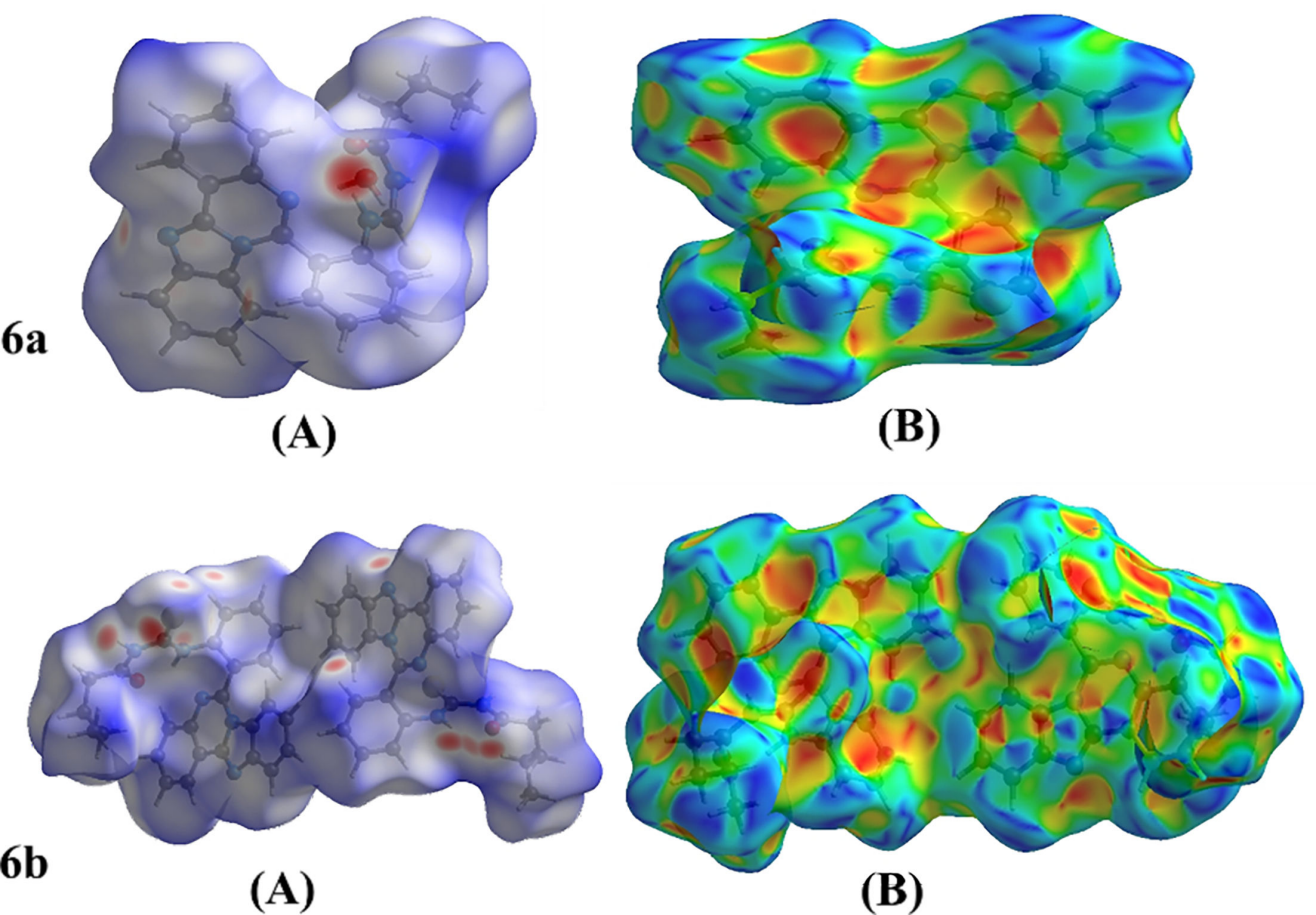
(A) View of the three-dimensional hirshfeld surface of the title compound plotted over d_norm_ in the range -0.6859 to 1.2036 a.u. (B) Three-dimensional Hirshfeld surface of the title chemical displayed across electrostatic potential energy ranging from -0.0500 to 0.0500 a.u., utilizing the STO-3G basis set at the Hartree–Fock theoretical level. The N—H ... O, O—H ... N, and C—H ... O hydrogen-bond donors and/or acceptors are shown as blue and red patches surrounding the atoms, indicating positive and negative potentials, respectively.

The asymmetric unit of 6**a** contains an unco-ordinated water molecule as shown in Figure 3, where it is linked to the mother molecule through the O—H…N hydrogen bond (Table 1). There is also an intramolecular O—H…, N hydrogen bond (Table 1). The planar, [*A* (C2—C7), *B* (N1/N2/C1/C2/C7/C8), *C* (N1/N3/C8/C9/C14), *D* (C9—C14) and *E* (C15—C20)], rings are oriented at dihedral angles of *A*/*B* = 3.91(6)°, *A*/*C* = 6.68(7)°, *A*/*D* = 7.84(6)°, *A*/*E* = 58.51(6)°, *B*/*C* = 4.48(7)°, *B*/*D* = 6.42(5)°, *B*/*E* = 60.21(5)°, *C*/*D* = 2.14(6)°, *C*/*E* = 64.60(6)° and *D*/*E* = 66.22(6)°. Consequently, the rings A and B, B and C, and C and D are nearly coplanar. Conversely, atom N4 is positioned 0.0159(18) Å from the corresponding ring E plane. Therefore, it is coplanar with the ring E. The crystal structure features intra- and intermolecular O—H···N, N—H···N, and N—H···O hydrogen bonds (Table 1) that connect the molecules into infinite chains along the b-axis direction (Figure 4). On the other hand, there are π···π interactions between the parallel *C* rings and parallel *D* rings [Cg1··· Cg1^i^ = 3.3288(13) Å and Cg4··· Cg4^ii^ = 3.6765(13) Å, where Cg1 and Cg4 are the centroids of rings, *C* (N1/N3/C8/C9/C14) and *D* (C9—C14), respectively. Symmetry codes: (i) –x, 2 – y, 1 – z; (ii) –x, 1 – y, 1 – z]. A weak C*—*H··· π interaction (Table 1) is also observed. All of these interactions are effective in the stabilization of the crystal structure.

#### Crystal structure of 6b

The asymmetric unit of the **6b** contains two crystallographically independent molecules (Figure 4). The planar, [*A* (C1—C6), *B* (N1/N2/C1/C2/C7), *C* (N2/N3/C7/C8/C13/C14), *D* (C8—C13) and *E* (C21—C26)] and [*A’* (C1a—C6a), *B’* (N1a/N2a/C1a/C2a/C7a), *C’* (N2a/N3a/C7a/C8a/C13a/C14a), *D’* (C8a—C13a) and *E’* (C21a—C26a)], rings are oriented at dihedral angles of [*A*/*B* = 2.32(8)°, *A*/*C* = 4.02(7)°, *A*/*D* = 4.30(8)°, *A*/*E* = 62.99(7)°, *B*/*C* = 1.71(8)°, *B*/*D* = 1.98(8)°, *B*/*E* = 61.98(7)°, *C*/*D* = 0.36(7)°, *C*/*E* = 61.53(6)° and *D*/*E* = 61.21(6)°] and [*A’*/*B’* = 1.92(8)°, *A’*/*C’* = 3.50(7)°, *A’*/*D’* = 4.33(8)°, *A’*/*E’* = 61.37 (7)°, *B’*/*C’* = 1.59(8)°, *B’*/*D’* = 2.41(8)°, *B’*/*E’* = 61.84 (8)°, *C’*/*D’* = 0.83(7)°, *C’*/*E’* = 62.08 (6)° and *D’*/*E’* = 62.25 (7)°]. Thus, [*A* and *B*, *A* and *C*, *A* and *D*, *B* and *C*, *B* and *D*, *C* and *D*] and [*A’* and *B’*, *A’* and *C’*, *A’* and *D’*, *B’* and *C’*, *B’* and *D’*, *C’* and *D’*] rings are almost coplanar. On the other hand, atom N4 is -0.0414 (17) Å away from the corresponding, *E* ring plane, while atom N4a is 0.1082 (19) Å away from the corresponding, *E’* ring plane. Thus, they are almost coplanar with the corresponding rings. In the crystal structure, intra- and intermolecular N—H···S and bifurcated N—H···O and N—H···N hydrogen bonds (Table 1) link the molecules into infinite double-chains, enclosing R_2_^2^(8) ring motifs (Figure 4). There are π···π interactions between the almost parallel *B* and *B’*, *B* and *C’* and parallel *A* rings [Cg1···Cg2^i^ = 3.4863 (13) Å, *α* = 0.85°; Cg1···Cg4^i^ = 3.7894 (13) Å, *α* = 0.90° and Cg6···Cg6^i^ = 3.9850 (13) Å, *α* = 0.03°, where Cg1, Cg2, Cg4, and Cg6 are the centroids of *B*, *B’*, *C’* and *A* rings, respectively. Symmetry code: (i) 1 — x, 1 — y, 1 — z]. On the other hand, a weak C*—*H··· π interaction (Table 1) is also observed. All of these interactions are effective in the stabilization of the crystal structure.

#### Hirshfeld surface analysis

The locations of atoms involved in short contacts with the potential to form hydrogen bonds, as well as the quantitative ratios of these interactions along with the π-stacking interactions, were examined using a Hirshfeld surface (HS) analysis using Crystal Explorer 17.5 in order to visualize the intermolecular interactions in the crystal of the title compound. The normalized contact distance, dnorm, aids in identifying locations that are especially crucial for intermolecular interactions. Red and blue colors indicate distances that are less (in close proximity) or longer (far contact) than the van der Waals radii, respectively, whereas the white surface in the HS plotted over the dnorm (Figure 3A) represents contacts with distances equal to the sum of the van der Waals radii.

Additionally, shape index was used to display the C—H···π interactions and the π···π stacking. The shape-index surface is utilized to identify unique packing modes, specifically planar stacking configurations and the presence of aromatic stacking interactions, such as C—H···π and π···π interactions. As for the C—H···π connections, the shape index describes them as ‘red p-holes’, referring to the centroids of the aromatic rings of neighboring molecules and the electron ring interactions between the CH groups.

**Figure 5:**
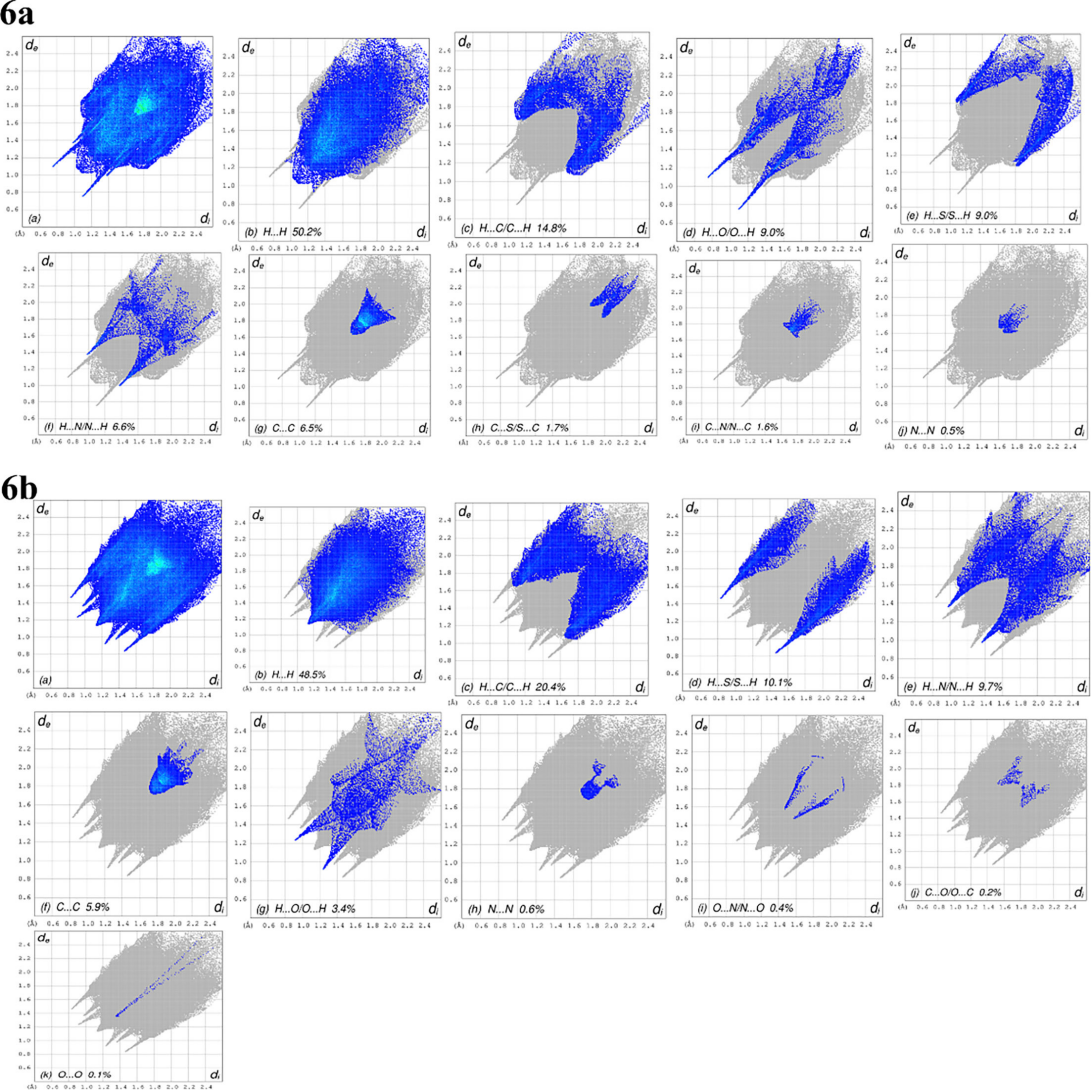
6a: The full two-dimensional fingerprint plots for the title compound, showing (a) all interactions, and delineated into (b) H···H, (c) H···C/C···H, (d) H···O/O···H, (e) H···S/S···H, (f) H···N/N···H, (g) C···C, (h) C···S/S···C, (i) C···N/N···C and (j) N···N interactions. The di and de values represent the nearest internal and external distances (in Å) from specified points on the Hirshfeld surface contacts. 6b: The full two-dimensional fingerprint plots for the title compound, showing (a) all interactions, and delineated into (b) H···H, (c) H···C/C···H, (d) H···S/S···H, (e) H···N/N···H, (f) C···C, (g) N···N, (H) H···O/O···H, (i) O···N/N···O, (j) C···O/O···C and (k) O···O interactions. The d_i_ and d_e_ values are the closest internal and external distances (in Å) from given points on the Hirshfeld surface contacts

**6a:** Figure 5a presents the overall two-dimensional fingerprint plot, while Figure 5b—j illustrates the delineated interactions: H···H, H···C/C···H, H···O/O···H, H···S/S···H, H···N/N···H, C···C, C···S/S···C, C···N/N···C, and N···N [25], along with their respective contributions to the Hirshfeld surface. The primary interaction is H···H (Table 2), accounting for 50.2% of the overall crystal packing. This is illustrated in Figure 5b, which shows widely scattered points of high density, attributable to the significant hydrogen content of the molecule, with the tip at d_e_ = d_i_ = 1.06 Å. In the context of C—H···π interactions (Table 3), the characteristic wings in the fingerprint plot were delineated into H···C/C···H contacts (Table 3 and Figure 5c), contributing 14.8% to the HS, with tips located at d_e_+d_i_ = 2.77 Å. The symmetrical pair of spikes in the fingerprint plot, characterized by H···O/O···H contacts (Table 3 and Figure 5d), contributes 9.0% to the HS, with the tips positioned at d_e_+d_i_ = 1.84 Å. The symmetrical pair of wings seen at the tips corresponds to d_e_+d_i_ = 2.87 Å, and the H···S/S···H contacts contribute 9.0% to the HS. The symmetrical pair of spikes of the H···N/N···H contacts (Table 3 and Figure 5f), contributing 6.6% to the HS, is observed with the tips at d_e_+d_i_ = 2.38 Å. However, when seen with the tip at d_e_ = d_i_ = 1.66 Å, the C···C contacts (Table 2 and Figure 5g) contain a bullet-shaped distribution of points that contribute 6.5% to the HS. In conclusion, the C···S/S···C (Table 2 and Figure 5h), C···N/N···C (Figure 5i), and N···N (Figure 5j) contacts exhibit low point densities, contributing 1.7%, 1.6%, and 0.5% to the HS, respectively. **6b:** Figure 4 clearly suggests that there are π···π interactions in (I). Figure 5a presents the overall two-dimensional fingerprint plot, while Figure 4b–k illustrates the plots categorized into H···N/N···H, C···C, N···N, H···H, H···C/C···H, H···S/S···H, H···O/O···H, C···O/O···C , O···N/N···O, and O···O, along with their respective contributions to the Hirshfeld surface. The primary interaction is H···H (Table 3), accounting for 48.5% of the overall crystal packing. This is illustrated in Figure 4b, which shows widely scattered points of high density, attributable to the significant hydrogen content of the molecule, with the tip at d_e_ = d_i_ = 1.14 Å. In the context of C—H··· π interactions (Table 3), the distinctive pair of wings in the fingerprint plot is divided into H···C/C···H contacts (Table 3 and Figure 5c, contributing 20.4% to the HS), with the tips positioned at d_e_+d_i_ = 2.71 Å. The contribution of the H···S/S···H contacts to the HS is 10.1%, with the symmetrical pair of spikes observed at tips corresponding to d_e_+d_i_ = 2.30 Å (Table 3 and Figure 5d). The symmetrical pair of spikes associated with the H···N/N···H contacts (Table 3 and Figure 5e), contributing 9.7% to the HS, is observed with the tips at d_e_+d_i_ = 2.34 Å. The C···C contacts (Table 3 and Figure 5f) exhibit a bullet-shaped distribution of points, contributing 5.9% to the HS, observed with the tip at d_e_ = di = 1.74 Å. The symmetrical pair of spikes in the fingerprint plot, characterized by H···O/O···H contacts (Table 3 and Figure 5g), contributes 3.4% to the HS, with the tips positioned at d_e_+d_i_ = 2.16 Å. In conclusion, the N···N (Table 3 and Figure 5h), O···N/N···O (Table 3 and Figure 5i), C···O/O···C (Figure 5j), and O···O (Table 3 and Figure 5k) contacts exhibit low point densities, contributing 0.6%, 0.4%, 0.2%, and 0.1% to the HS, respectively.

**Table 2:** The HOMO and LUMO values calculated for DFT and TD-DFT calculations, respectively (6a–h)

Codes	Method	HOMO (eV)	LUMO (eV)	HOMO-LUMO ( ∆ eV)
**6a**	DFT	-0.207	-0.065	0.142
	TD-DFT	-0.199	-0.075	0.120
**6b**	DFT	-0.208	-0.066	0.142
	TD-DFT	-0.199	-0.075	0.121
**6c**	DFT	-0.205	-0.065	0.14
	TD-DFT	-0.198	-0.076	0.122
**6d**	DFT	-0.202	-0.080	0.122
	TD-DFT	-0.199	-0.074	0.122
**6e**	DFT	-0.203	-0.080	0.123
	TD-DFT	-0.199	-0.076	0.120
**6f**	DFT	-0.202	-0.081	0.121
	TD-DFT	-0.199	-0.075	0.120
**6g**	DFT	-0.202	-0.080	0.122
	TD-DFT	-0.197	-0.074	0.123
**6h**	DFT	-0.200	-0.080	0.12
	TD-DFT	-0.198	-0.074	0.121

**Table 3:** Selected Interatomic distances (Å) for 6a and 6b

6a				6b			
**S1···C17**	3.216 (2)	**C13···C15**	3.249 (3)	**S1···H11^i^**	2.95	**C2···C26**	3.396 (3)
**S1···H17**	2.86	**C13···C20**	3.280 (3)	**S1···H23**	2.71	**C2A···C26A**	3.384 (3)
**H1WB···S1^i^**	2.97 (5)	**C14···C20**	3.393 (3)	**H5A···S1^ii^**	2.40 (2)	**C3···C26**	3.205 (3)
**O1···N4**	2.645 (2)	**C1···H4**	2.70 (3)	**S1···H12^iii^**	2.94	**C3···C21**	3.242 (3)
**O1W···N5^i^**	2.872 (3)	**C15···H13**	2.74	**H17D···S1^ii^**	2.80	**C3A···C26A**	3.206 (3)
**O1···H4**	1.89 (3)	**C20···H13**	2.74	**S1···H23^iv^**	2.80	**C3A···C21A**	3.251 (3)
**O1···H23A^ii^**	2.60	**C21···H17**	2.89	**H5···S1A^v^**	2.66 (2)	**C14A···C15A**	3.379 (3)
**H5···O1W^iii^**	2.00 (3)	**C22···H4**	2.39 (3)	**O1···N4**	2.635 (3)	**C14···H4**	2.57 (3)
**N2···N4**	3.010 (2)	**H1WA···H5^i^**	2.37 (5)	**O1···N3**	3.079 (3)	**C15···H23**	2.89
**N2···H1WA**	1.94 (3)	**H1WB···H5^i^**	2.28 (6)	**O1A···N4A**	2.653 (3)	**C16···H19B**	2.70
**N2···H4**	2.48 (3)	**H5···H23B**	2.14	**O1A···O1A^vi^**	2.727 (3)	**C16···H4**	2.40 (3)
**N3···H20^iv^**	2.51	**H17···H6^v^**	2.25	**O1···H4**	1.88 (2)	**C16A···H19C**	2.88
**H18···N3^v^**	2.71			**O1···H18B**	2.63	**C16A···H4A**	2.46 (3)
				**O1A···H4A**	1.98 (3)	**C20···H17B**	2.80
				**O1A···H4A^vi^**	2.29 (2)	**C21···H3**	2.73
				**N3···N4**	2.931 (3)	**C21A···H3A**	2.75
				**N3A···N4A**	3.045 (3)	**C26···H3**	2.60
				**N4···N3**			2.62
				**H26A···N1^i^**			2.21
				**H26···N1A^vii^**			2.21
				**N3···H4**	2.32 (2)		

The nearest neighbor co-ordination environment of a molecule can be determined from the color patches on the HS based on how close to other molecules they are. The Hirshfeld surface representations with the function d_norm_ plotted onto the surface are shown for the H···H, H···C/C···H, H···S/S···H, and H···N/N···H interactions in Figure 5a—d, respectively.

The analysis of the Hirshfeld surface confirms the significance of hydrogen atom contacts in determining the packing arrangement. The significant presence of H···H, H···C/C···H, H···S/S···H, and H···N/N···H interactions indicates that van der Waals interactions and hydrogen bonding are primary contributors to the crystal packing [26].

The analysis of the Hirshfeld surface supports the significance of hydrogen atom interactions in determining the packing arrangement. The substantial presence of H···H, H···C/C···H, H···O/O···H, and H···S/S···H interactions indicates that van der Waals forces and hydrogen bonding are pivotal in the arrangement of the crystal structure [25].

Symmetry codes: (i) *x* + 1, *y*, *z*; (ii) *x*, *y* + 1, *z*; (iii) −*x*, −*y*, −*z* + 1; (iv) −*x* + 1, −*y*, −*z* + 1; (v) *x*, *y* - 1, *z*; (vi) −*x* + 1, −*y* + 2, −*z*; (vii) *x*−1, *y*, *z*.

**Figure 6:**
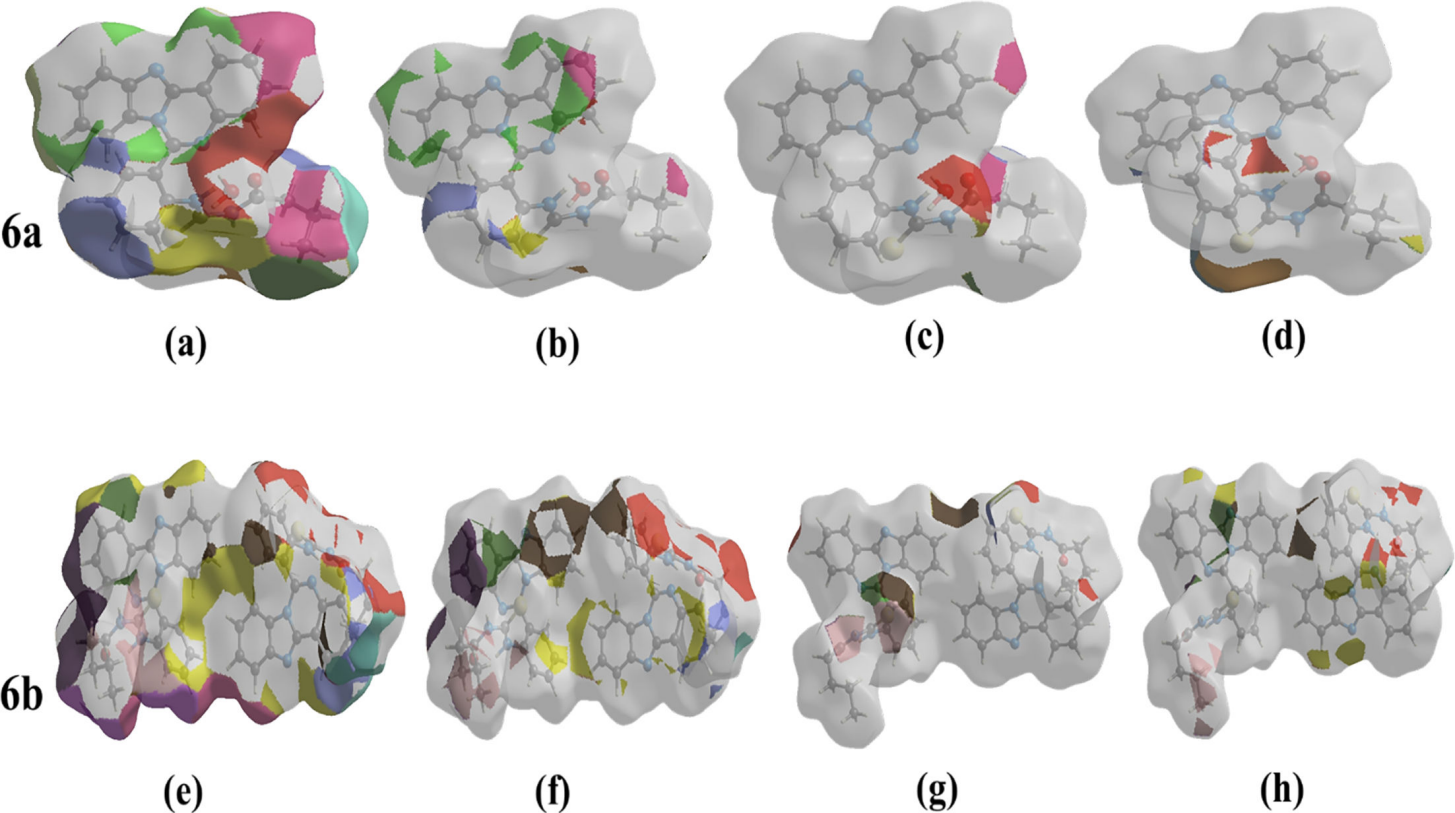
The Hirshfeld surface representations with the function d_norm_ plotted onto the surface for (a) and (e) H···H, (b) and (f) H···C/C···H, (c) H···O/O···H, (d) H···S/S···H, (G) H···S/S···H and (h) H···N/N···H interactions.

The color regions on the HS can be used to determine the nearest neighbor co-ordination environment of a molecule by comparing their proximity to other molecules. The Hirshfeld surface representations for the H···H, H···C/C···H, H···O/O···H, and H···S/S···H interactions are exhibited in Figure 6a—d for compound **6a** and Figure 6e–h for compound **6b**, respectively, with the function d_norm_ plotted onto the surface.

The analysis of the Hirshfeld surface validates the significance of hydrogen atom contacts in determining the packing arrangement. The significant presence of H···C/C···H, H···H, H···O/O···H, and H···S/S···H interactions indicates that van der Waals interactions and hydrogen bonding are the primary contributors to the crystal packing [25].

### Density functional theory (DFT)

#### Molecular geometry

Time-dependent density-functional theory (TDDFT) extends the principles of ground-state density-functional theory (DFT) to include excitations and various time-dependent phenomena. DFT was used to obtain ground-state descriptors (HOMO–LUMO gap, softness, polarizability) relevant to intrinsic reactivity, while TD-DFT was applied to describe excited-state charge-transfer characteristics. Using both methods provides a more complete electronic profile, strengthening the mechanistic rationale for the observed bioactivity [27,28]. The HOMO−LUMO band gap (Table 2) is an essential parameter in determining atomic electrical transport features, including the chemical reactivity of a material and the kinetic stability of a molecule, since it quantifies electron conductivity [29].

**Figure 7:**
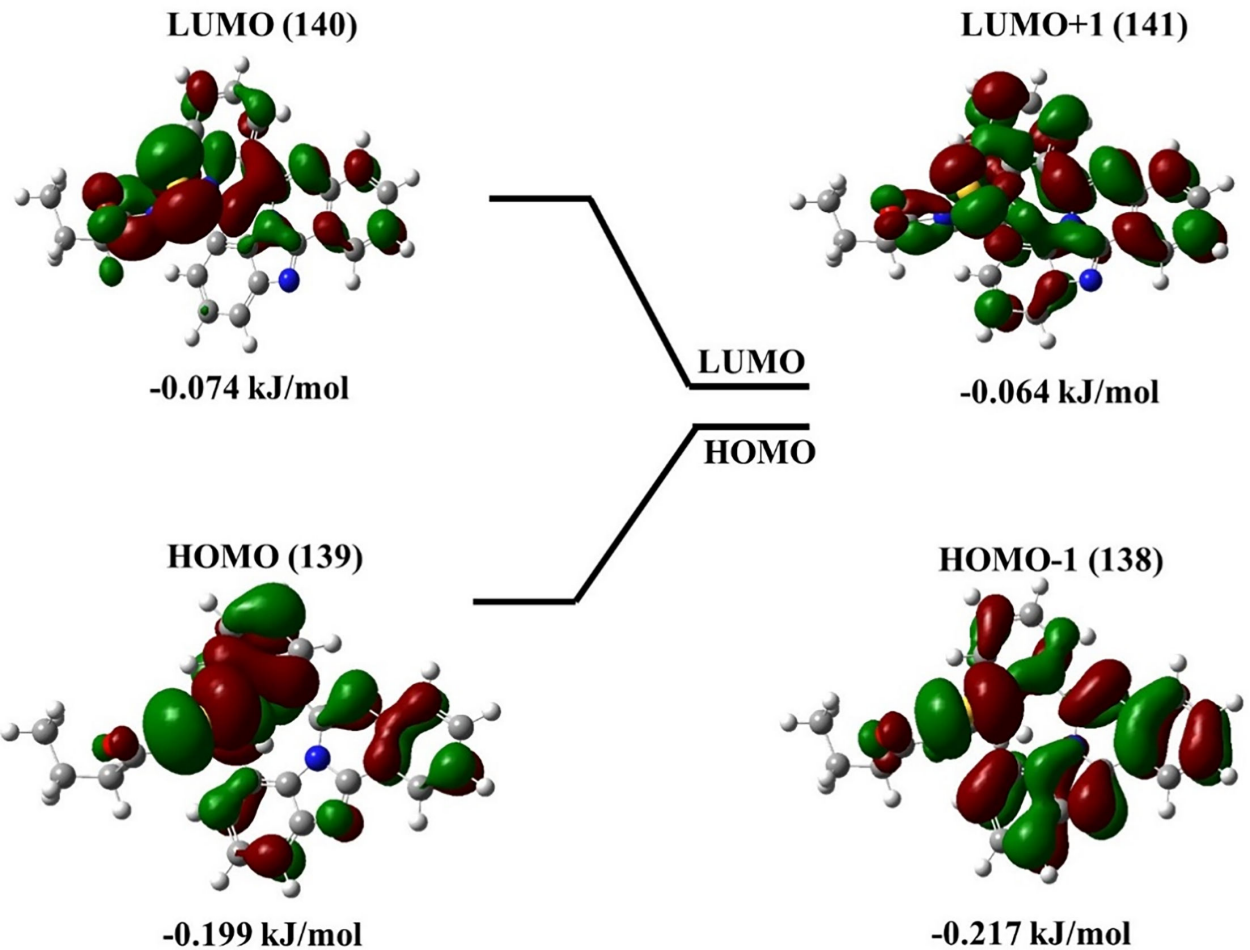
Compound 6g with HOMO, LUMO, HOMO-1, and LUMO+1 structures

The HOMO, as well as the LUMO of the potent compound **6g** according to AutoDock Vina, is distributed over the entire molecule, with LUMO having more antibonding characteristics. The calculated energy gap is 0.123 eV. 3D plots of the HOMO − 1, HOMO, LUMO, and LUMO + 1 orbitals computed at the B3LYP/6–311G (d,p) level for **6g** molecule are illustrated in Figure 7.

The optimized structures are given in Supplementary file.

Chemical potential in the electronic binding energies of an atom in different chemical environments can be used to derive information on the chemical bonding in the different environments [30]. Polarizability has a direct correlation with the binding tendency of the ligand; a highly polarizable ligand is conceivable to bind more strongly to its target as compared with a weakly polarizable ligand. It was found that **6g** had the highest polarizability compared with other compounds. The dipole moment in a molecule is another important electronic property. For example, the bigger the dipole moment, the stronger the intermolecular interactions will be. Electronegativity (χ) is the negative chemical potential which describes the proclivity of electrons to leave a stable system, while chemical hardness is a parameter to measure the resistance to alter the electron distribution and hence can be associated with the reactivity of the chemical system [31]. The results given in Table 4 show that the synthesized compounds are chemically reactive and soft in nature.

**Table 4:** Chemical parameters calculated in accordance with DFT and TD-DFT methods of compounds 6a–h

Codes	Method	Optimization energy	Dipole moment	Polarizability (α)	Chemical potential µ (eV)	Electronegativity X (eV)	Hardness ƞ (eV)	Softness S (eV-1)
**6a**	DFT	-1712.149	7.619	323.266	-0.136	0.136	0.071	7.042
	TD-DFT	-1711.761	5.386	----------	-0.137	0.137	0.062	8.065
**6b**	DFT	-1751.453	7.600	334.589	-0.137	0.137	0.071	7.042
	TD-DFT	-1751.065	5.343	----------	-0.137	0.137	0.062	8.065
**6c**	DFT	-1790.758	7.573	366.651	-0.135	0.135	0.070	7.143
	TD-DFT	-1790.368	5.317	----------	-0.137	0.137	0.061	8.197
**6d**	DFT	-1830.064	7.366	372.993	-0.141	0.141	0.061	8.197
	TD-DFT	-1829.671	5.297	----------	-0.137	0.137	0.063	8.000
**6e**	DFT	-1869.365	7.291	383.347	-0.142	0.142	0.062	8.130
	TD-DFT	-1868.971	5.239	----------	-0.138	0.138	0.062	8.130
**6f**	DFT	-1908.669	7.300	393.991	0.142	0.142	0.061	8.264
	TD-DFT	-1908.274	5.269	----------	0.137	0.137	0.062	8.065
**6g**	DFT	-1947.972	7.276	404.325	0.141	0.141	0.061	8.197
	TD-DFT	-1947.577	5.247	----------	0.136	0.136	0.062	8.130
**6h**	DFT	-1751.456	7.533	349.598	0.140	0.140	0.060	8.333
	TD-DFT	-1751.058	6.215	----------	0.136	0.136	0.062	8.065

These values suggest that compound **6g** showed higher polarizability and softness values compared with other compounds, which proves its reactivity and chemical stability.

### Structure–activity relationships (SAR)

A series of acylthiourea derivatives (**6a–6h**) were synthesized by incorporating various alkyl chains into the isocryptolepine ‘aza’ core, and their inhibitory potential was assessed against tyrosinase, carbonic anhydrase, and alkaline phosphatase. Overall, the SAR analysis reveals a strong dependence of biological activity on the nature and length of the alkyl substituents. All compounds displayed excellent to moderate inhibition of tyrosinase and carbonic anhydrase, while their effects on alkaline phosphatase were comparatively modest. Notably, compound **6g**, bearing a decyl chain, consistently emerged as the most potent inhibitor across all three enzymes, highlighting the importance of extended hydrophobic interactions in enhancing target binding.

The SAR analysis of the synthesized compounds reveals that subtle structural modifications significantly affect their biological activity and binding affinities. The introduction of electron-donating and electron-withdrawing substituents on the aromatic ring demonstrates a clear influence on potency, where electron-withdrawing groups (e.g. halogens, nitro) enhance target binding through increased polarity and potential hydrogen-bond acceptor capacity, while electron-donating groups improve lipophilicity. Furthermore, the incorporation of heteroatoms such as nitrogen, oxygen, or sulfur in the core scaffold facilitates additional noncovalent interactions (hydrogen bonding, dipole–dipole, or π–π stacking), thereby stabilizing the ligand–receptor complex. The length and flexibility of linkers play a decisive role in maintaining an optimal spatial orientation of pharmacophores, ensuring better complementarity with the binding pocket. Bulky substituents, although enhancing hydrophobic interactions, may sometimes hinder binding due to steric clashes, highlighting the importance of balanced substitution. Overall, the SAR indicates that fine-tuning of electronic effects, steric properties, and hydrogen-bonding functionalities in the designed framework provides an effective strategy to improve specificity, selectivity, and therapeutic potential of the compounds.

**Figure 8:**
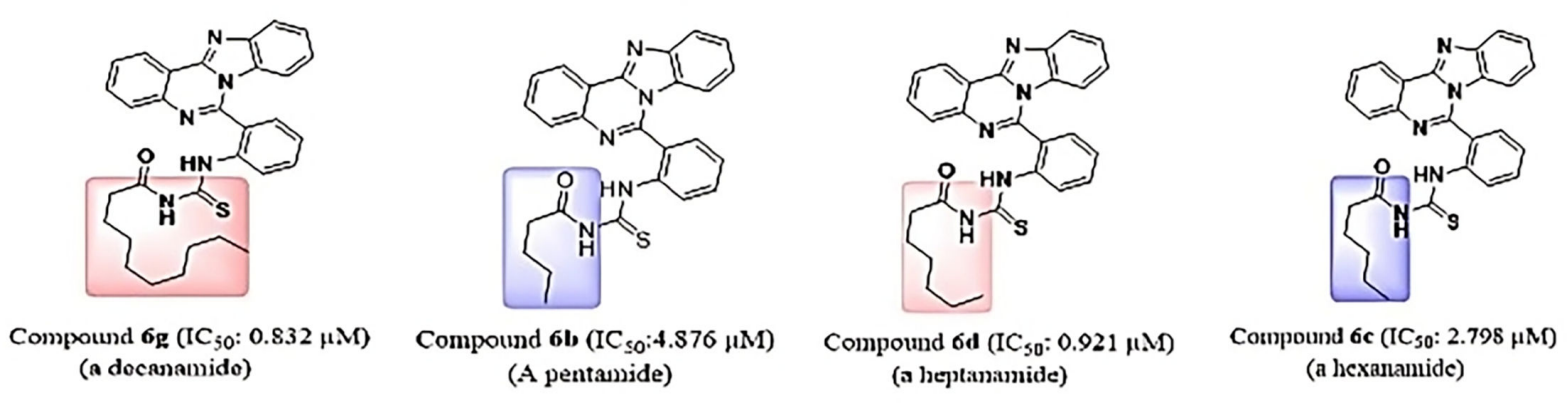
Comparison of tyrosinase inhibitory activity of long vs. short chain containing compounds.

For tyrosinase inhibition, all derivatives significantly outperformed the standard kojic acid (IC₅₀ = 16.832 µM), with IC₅₀ values ranging from 0.832 to 7.945 µM. Longer alkyl chains enhanced activity, as exemplified by **6g** (0.832 µM, decyl) and **6d** (0.921 µM, heptyl) compared with shorter chain analogs **6b** (4.876 µM, pentyl) and **6c** (2.798 µM, hexyl). Similarly, the octyl-substituted **6e** (1.846 µM) demonstrated strong inhibition, while the introduction of a bulky tert-butyl group in **6h** (1.273 µM) also yielded high potency, suggesting that both extended hydrophobic chains and steric bulk contribute favorably to tyrosinase inhibition (Figure 8).

**Figure 9:**
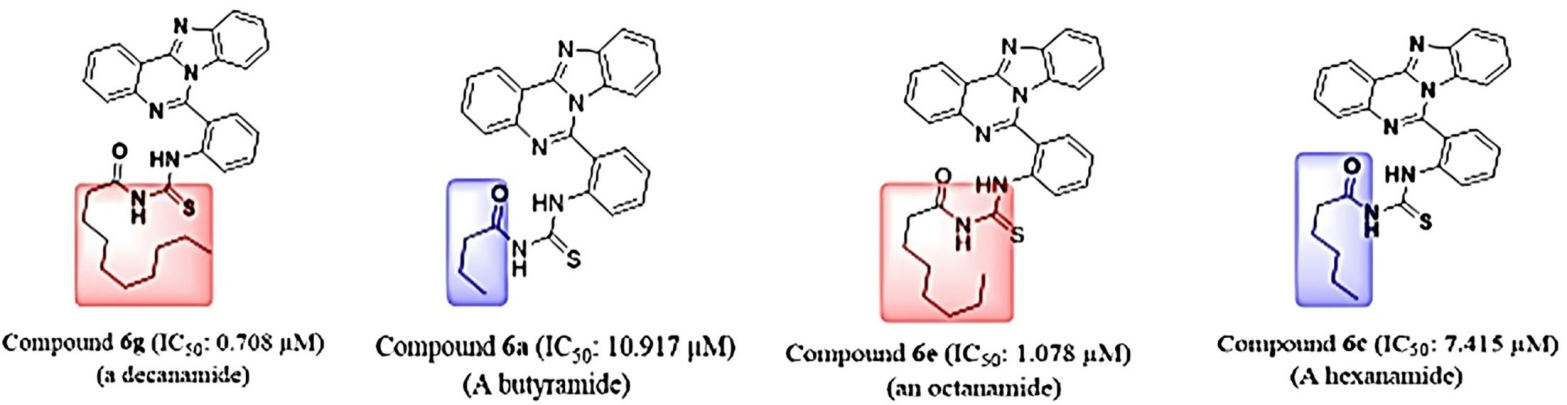
Comparison of carbonic anhydrase inhibitory activity of long vs. short chain containing compounds.

In the case of carbonic anhydrase, the inhibitory profile of these derivatives was superior to the standard oleanolic acid, with a clear trend favoring longer alkyl chains. Compound **6g** (0.708 µM) was again the most active, while **6a** (10.917 µM, butyl) was the weakest. The activity comparison between **6e** (1.078 µM, octyl) and **6c** (7.415 µM, hexyl) supports this length-dependent enhancement. Interestingly, exceptions were noted: **6f** (nonyl, 7.141 µM) and **6d** (heptyl, 0.309 µM) deviated from the trend, indicating that optimal chain length and spatial orientation rather than mere elongation determine binding efficacy. Compound **6h** (tert-butyl, 2.331 µM) also displayed excellent activity, further underscoring the role of steric and electronic effects in modulating enzyme interactions (Figure 9).

**Figure 10:**
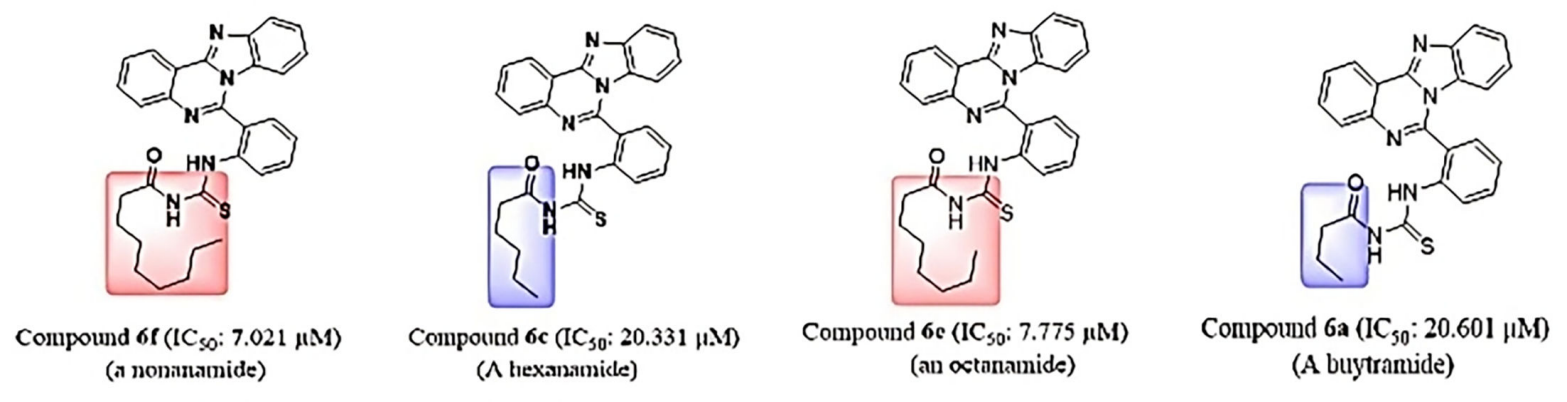
Comparison of alkaline phosphatase inhibitory activity of long vs. short chain containing compounds.

Against alkaline phosphatase, most compounds demonstrated only moderate inhibition (IC₅₀ = 7.021–20.601 µM) compared with the KH₂PO₄ standard, with **6g** (2.812 µM) again standing out as the only compound surpassing the reference (Table 5). The results suggest that extended alkyl chains enhance hydrophobic interactions within the enzyme’s binding site, thereby improving activity. The tert-butyl derivative **6h** also retained good potency, reaffirming the beneficial impact of bulky substituents (Figure 10).

**Table 5:** Tyrosinase, carbonic anhydrase, and alkaline phosphatase IC_50_ results of (6a–h).

Compound	Tyrosinase IC_50_ ± SEM (µM)	**Carbonic anhydrase** IC_50_ ± SEM (µM)	Alkaline phosphatase IC_50_ ± SEM (μM)
**6a**	7.945 ± 0.63	10.917 ± 0.99	20.61 ± 1.23
**6b**	4.876 ± 0.54	2.759 ± 0.56	10.719 ± 0.96
**6c**	2.798 ± 0.85	7.415 ± 0.74	20.33 ± 1.09
**6d**	0.921 ± 0.07	3.512 ± 0.63	16.59 ± 0.98
**6e**	1.846 ± 0.886	1.078 ± 0.42	7.77 ± 0.85
**6f**	2.051 ± 0.97	7.141 ± 0.95	7.0217 ± 0.61
**6g**	**0.832 ± 0.03**	**0.708 ± 0.02**	**2.812 ± 0.42**
**6h**	1.273 ± 0.17	2.331 ± 0.34	7.201 ± 0.63
**Kojic acid**	**16.83 ± 1.162**	**-----------------**	**------------------**
**Oleanolic acid**	**------------------**	**12.841 ± 0.511**	**------------------**
**KH_2_PO_4_**	**------------------**	**------------------**	**5.241 ± 0.471**

Values are presented as Mean ± SEM (Standard error of the mean).

The SAR study identifies the isocryptolepine ‘aza’ core as the primary pharmacophore, with variations in alkyl substituents significantly affecting activity. Extended alkyl chains (C8–C10) increased efficacy against tyrosinase and carbonic anhydrase, aligning with enhanced hydrophobic interactions within the enzyme active sites. Compound **6g**, featuring a decyl chain, was identified as the most effective multi-target inhibitor, indicating that increased lipophilicity enhances triple-enzyme inhibition. The tert-butyl substituent in **6h** enhanced activity, probably due to steric hindrance and van der Waals interactions, but with less consistency compared with linear chains. In contrast, medium-chain analogs like **6d** and **6f** exhibited reduced activity, likely attributable to suboptimal chain orientation. The optimal pharmacophore consists of an aza core connected to a sufficiently long hydrophobic chain, a conclusion corroborated by studies on structurally related tyrosinase and CA inhibitors. The findings delineate an optimal pharmacophore characterized by the isocryptolepine Aza-core scaffold, enhanced by an extended hydrophobic chain, which imparts multi-target potency and identifies compound **6g** as the lead molecule in the series.

Taken together, these findings establish a clear SAR: longer alkyl chains generally confer enhanced enzyme inhibition, with steric bulk (tert-butyl) also contributing favorably in specific cases. The consistent superiority of compound **6g** highlights the importance of hydrophobic complementarity in binding, while the deviations observed in **6d** and **6f** suggest that structural orientation and conformational flexibility must also be considered.

According to the DFT and TD-DFT analyses, compound **6g** has the highest polarizability and softness among the series, as well as the smallest HOMO–LUMO gap (0.123 eV), which may be a factor in its superior multi-target inhibitory activity (Table 5). Increased polarizability may improve binding affinity by strengthening induced-dipole interactions within enzyme active sites, whereas a reduced HOMO–LUMO gap is typically linked to enhanced chemical reactivity, which could promote significant interactions with catalytic residues.

### Molecular docking

The molecular docking was done using the AutoDock Vina [32] software and the results were visualized by Pymol 2.0 Software [33]. The docking score is shown in Table 6. The results of molecular docking indicated that compound **6g** interacted significantly with all three proteins and intercalated DNA strongly. Tyrosinase was significantly involved in both hydrogen bonding and hydrophobic interactions, with a top docking score of -38 kJ/mol (Table 6), indicating that **6g** has the potential to inhibit this protein. These findings are in accordance with in vitro assays. Kojic acid, Levamisole, and Acetazolamide are used as controls for tyrosinase, alkaline phosphatase, and carbonic anhydrase II, respectively. For a more comprehensive understanding of the molecular interactions of the standard drugs, please refer to the Supplementary file.

**Table 6:** Binding scores of 6a–h within the active pocket of tyrosinase, alkaline phosphatase, and carbonic anhydrase with their respective controls

Docking score (kJ/mol)			
Compounds	Tyrosinase	Alkaline phosphatase	Carbonic anhydrase
6a	-36.4	-28.8	-27.6
6b	-35.6	-26.8	-27.2
6c	-34.8	-26.8	-28
6d	-34	-27.6	-28.4
6e	-31.2	-28	-28.8
6f	-36	-24.8	-31.2
6g	-**38**	-30.4	-26.4
6h	-36	-30.2	-30.4
Kojic acid	-22.4	______	______
Levamisole	______	-22.8	______
Acetazolamide	______	______	-27.6

The docking scores were largely aligned with the experimental inhibitory activities presented in Table 6. Compounds demonstrating stronger predicted binding affinities, including **6g**, **6d**, and **6e**, also showed the lowest IC₅₀ values among the three enzymes. In contrast, weaker binders such as **6a** and **6c** were associated with diminished potency. **6g** was identified as the most effective multi-target inhibitor, exhibiting IC₅₀ values of 0.832 µM for tyrosinase, 0.708 µM for carbonic anhydrase (CA), and 2.812 µM for alkaline phosphatase (ALP), significantly surpassing the reference standards. The favorable docking scores corresponded with the exceptional activity profile, indicating that **6g** effectively inhibits through stable hydrophobic and hydrogen-bonding interactions within the active sites. The close alignment of computational and experimental data enhances confidence in the docking model and substantiates the proposed pharmacophore design.

### Interactions of 6g with tyrosinase

**Figure 11:**
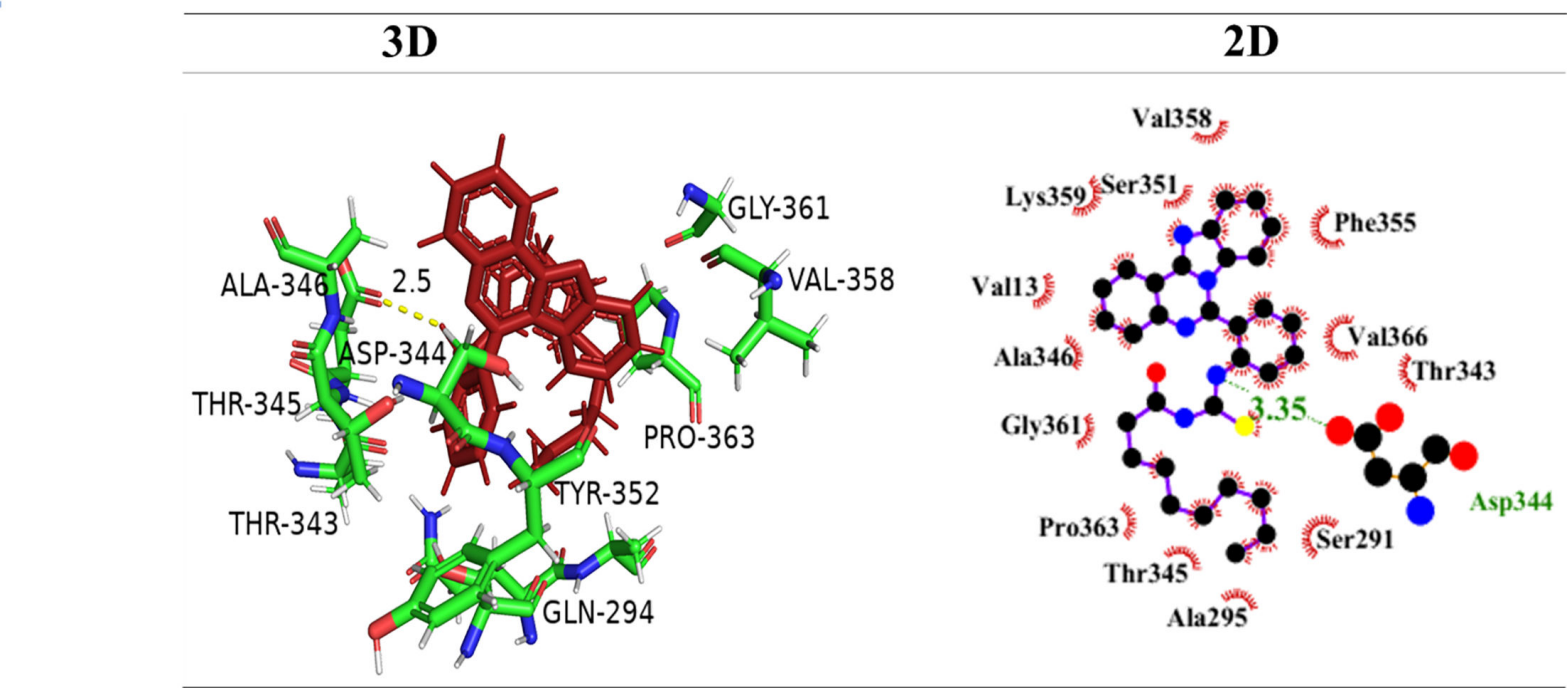
3D and 2D interactions of 6g within the active pocket of tyrosinase.

The molecular docking analysis of the interaction between tyrosinase and **6g** revealed a significant docking score of -38 kJ/mol. The hydrogen bonding residue involved in the interaction was ASP344 with hydrogen bond length of 2.5 Å (Figure 11). The hydrophobic interaction residues were VAL366, ALA346, GLN294, LYS359, THR345. These results suggest that compound **6g** can potentially inhibit the activity of tyrosinase, a key enzyme involved in the production of melanin in skin cancer, by forming stable hydrogen bonds with the protein’s key residues and forming hydrophobic interactions with other important residues. Figure 10 is illustrating the putative 2D and 3D binding interactions of tyrosinase with **6g**.

**Figure 12:**
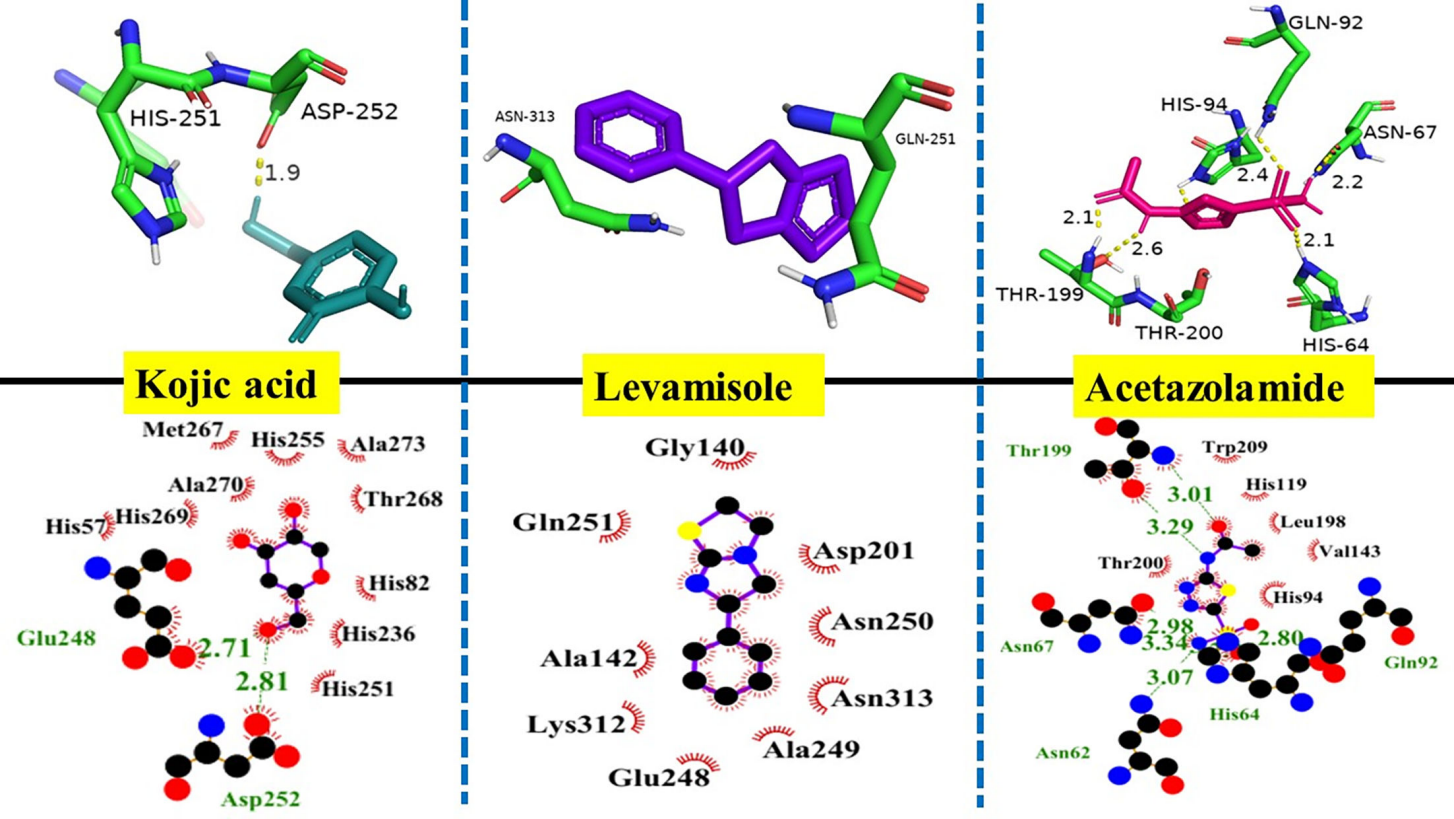
3D and 2D interactions of controls kojic acid, levamisole, acetazolamide with the active pocket of tyrosinase, alkaline phosphatase, and carbonic anhydrase II, respectively.

All controls showed less docking score compared with **6g** and other compounds. The 3D and 2D interactions of kojic acid given in Figure 12 indicate hydrogen bond interaction of amino acids ASP252 and GLU248 with bond angles of 1.9 and 2.7, respectively. They indicate a docking score of kJ/mol and fewer hydrophobic interactions as compared with the potent compound in our findings. Similarly, Levamisole, a control for Alkaline Phosphatase, showed no strong hydrophobic or ionic interactions with Alkaline phosphatase. Levamisole exhibited a docking score of -22.8 kJ/mol which is relatively low as compared with **6g**. Acetazolamide was taken as control for Carbonic Anhydrase II and showed a docking score of -27.6 kJ/mol and made strong hydrogen bond interactions with THR199, ASN67, ASN62, HIS64, respectively. The results indicate that currently used inhibitors are not sufficient to produce desired inhibition to indicate anticancer activity, and there is a need to develop potent and less toxic inhibitors.

### Molecular dynamics simulations

**Figure 13:**
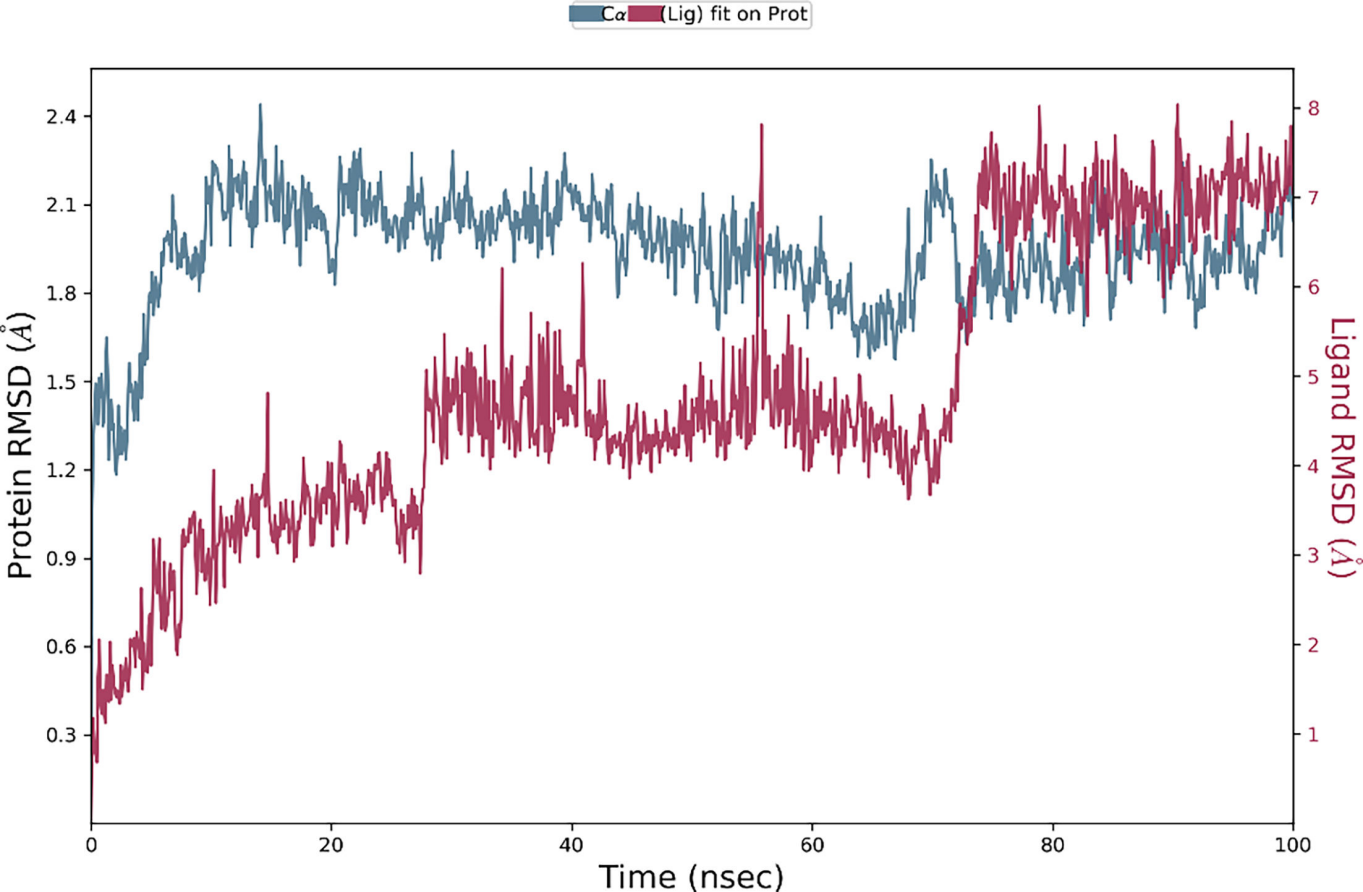
The evolution of RMSD pattern for protein-ligand complex and apo protein.

The top-ranked conformations of compound **6g** were subjected to molecular dynamics simulation concerning the tyrosinase enzyme. The docking simulation revealed that compound **6g** displayed strong binding interactions with tyrosinase. Molecular dynamics simulations were utilized to assess the stability and resilience of the interactions between the two drugs and the biological target. Figure 13 depicts the simulated trajectories of the tyrosinase-6g complex alongside the apo proteins. The comprehensive analysis of the stability profile of apo proteins reveals a notable stability trend over the simulation period. The robust strength of the molecular interactions involved is reflected in the finding of tyrosinase slightly varying at an RMSD value of about 2.4 angstroms. The computed average RMSD of the tyrosinase protein is 2.8 angstroms. The results indicate a reduced conformational change of the tyrosinase protein throughout the 100 ns simulation period. Ultimately, the substantial torsions of compound **6g** were instrumental in stabilizing the complex and reinforcing interactions.

**Figure 14:**
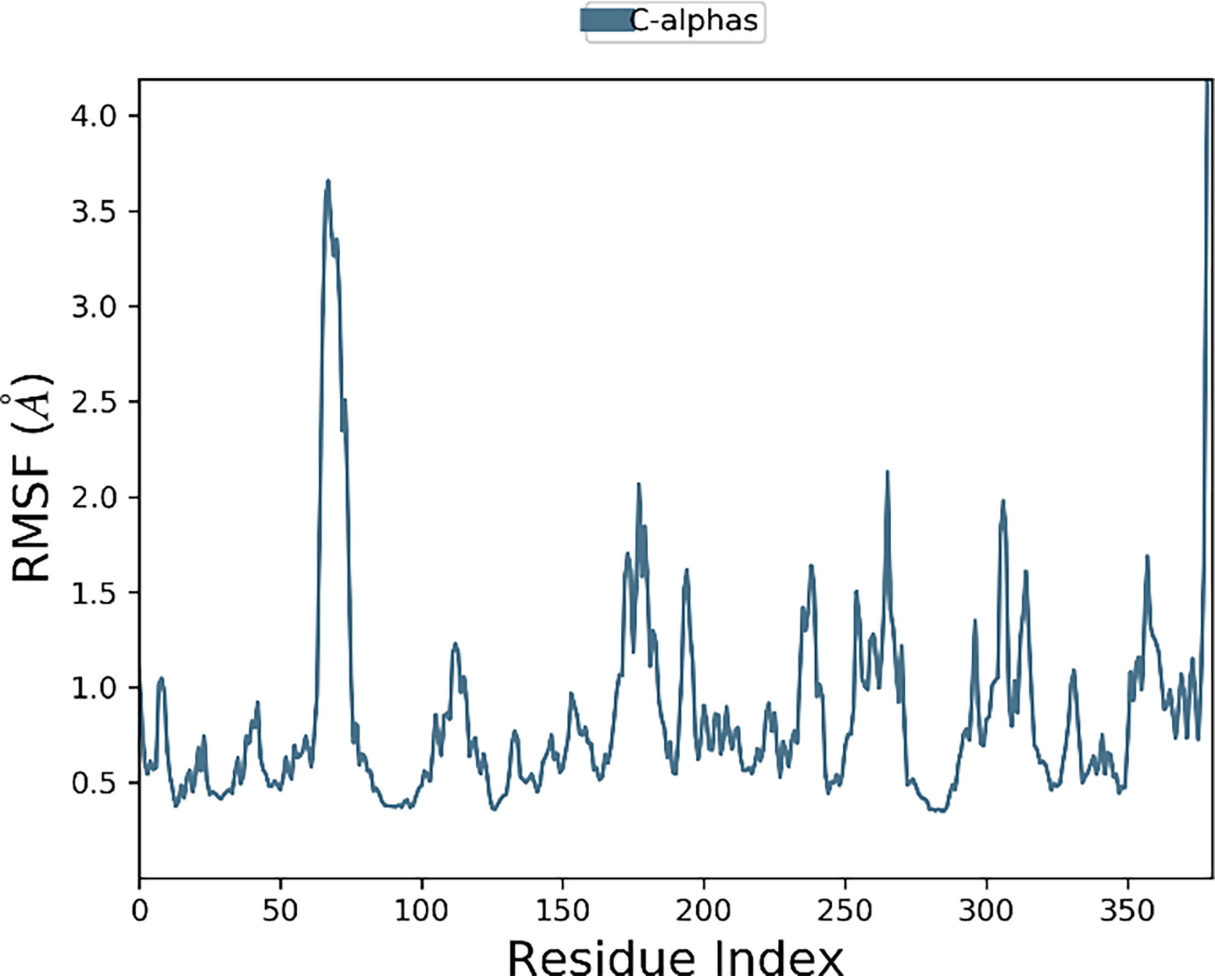
RMSF pattern for tyrosinase.

The calculations of residue variations were performed using the RMSF (root mean square fluctuations) metric. The amino acid residues exhibited optimal fluctuations, consistently staying under 2 angstroms. Nevertheless, the residues at the C and N terminals exhibited relatively higher levels of variability. This observation indicates that the ligand’s binding contributed to the stability of the rearrangements of these residues. Figure 14 displays the progression of RMSF for tyrosinase.

**Figure 15:**
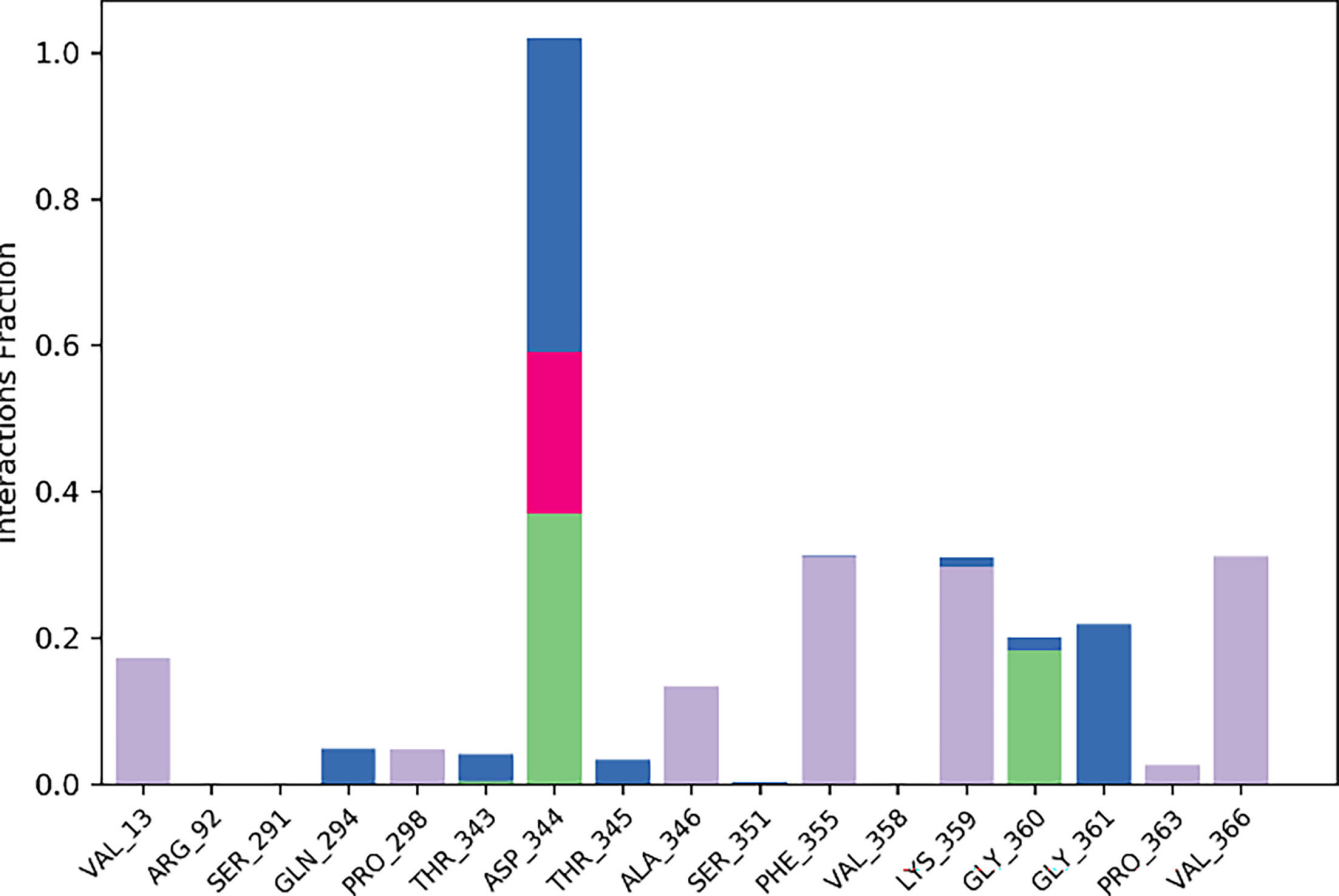
Contact map profile of protein-ligand complex.

The contact map profile illustrates the different hydrophilic and hydrophobic interactions that play a role in the stability of the protein-ligand complex. Compound **6g** exhibited significant interaction with the amino acid residues of tyrosinase. The key amino acid residues of tyrosinase that participate in hydrogen bonding are ASP344 and GLY360, respectively. The interaction fraction represented more than 10% of the total simulation duration. Furthermore, compound **6g** interacted with VAL13, PRO298, PHE355, and VAL366 via hydrophobic interactions. The contact map profile of tyrosinase-6g complex is illustrated in Figure 15.

**Figure 16:**
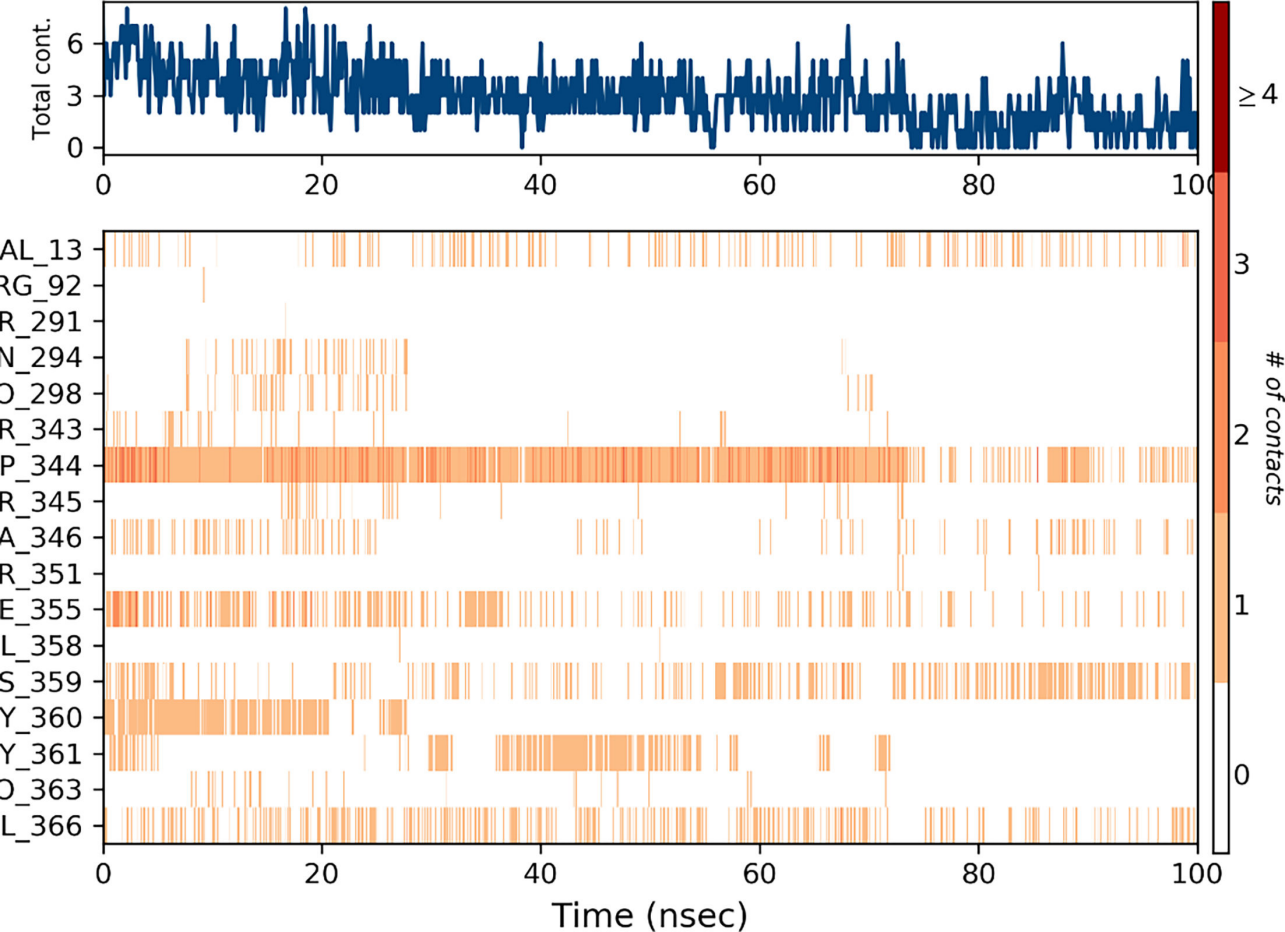
Contact map of **tyrosinase-6g** complex.

The contact map of complex (Figure 16) was also retrieved from simulated trajectory. The fewer interactions are represented by white or light colors, whereas strong interactions are indicated by dark colors.

**Figure 17:**
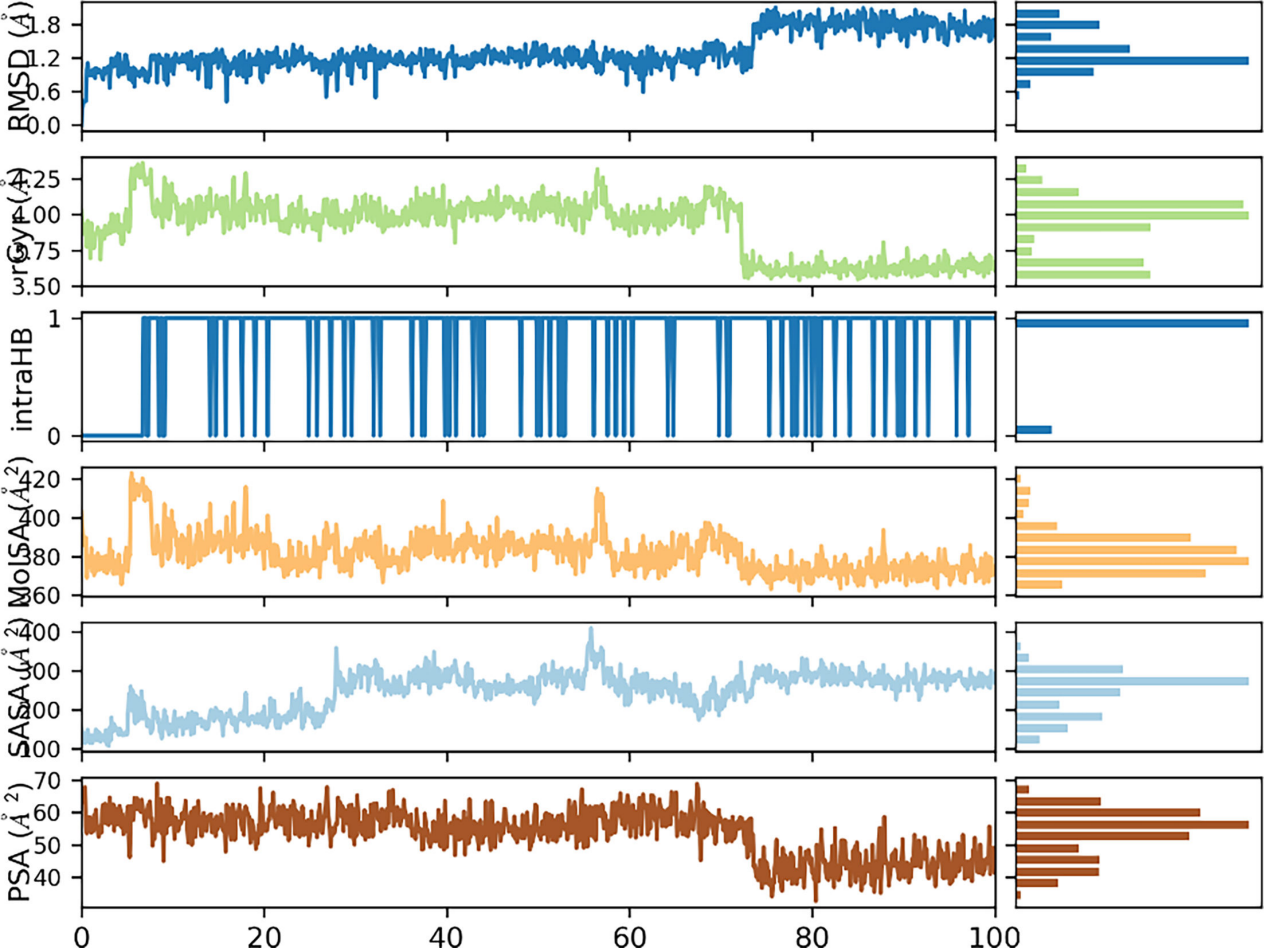
Dynamic parameters for tyrosinase-6g complex.

Important parameters including ligand properties were also computed from 100 ns simulated trajectory. Important parameters and ligand properties as a function of simulated time are shown in Figure 17.

**Figure 18:**
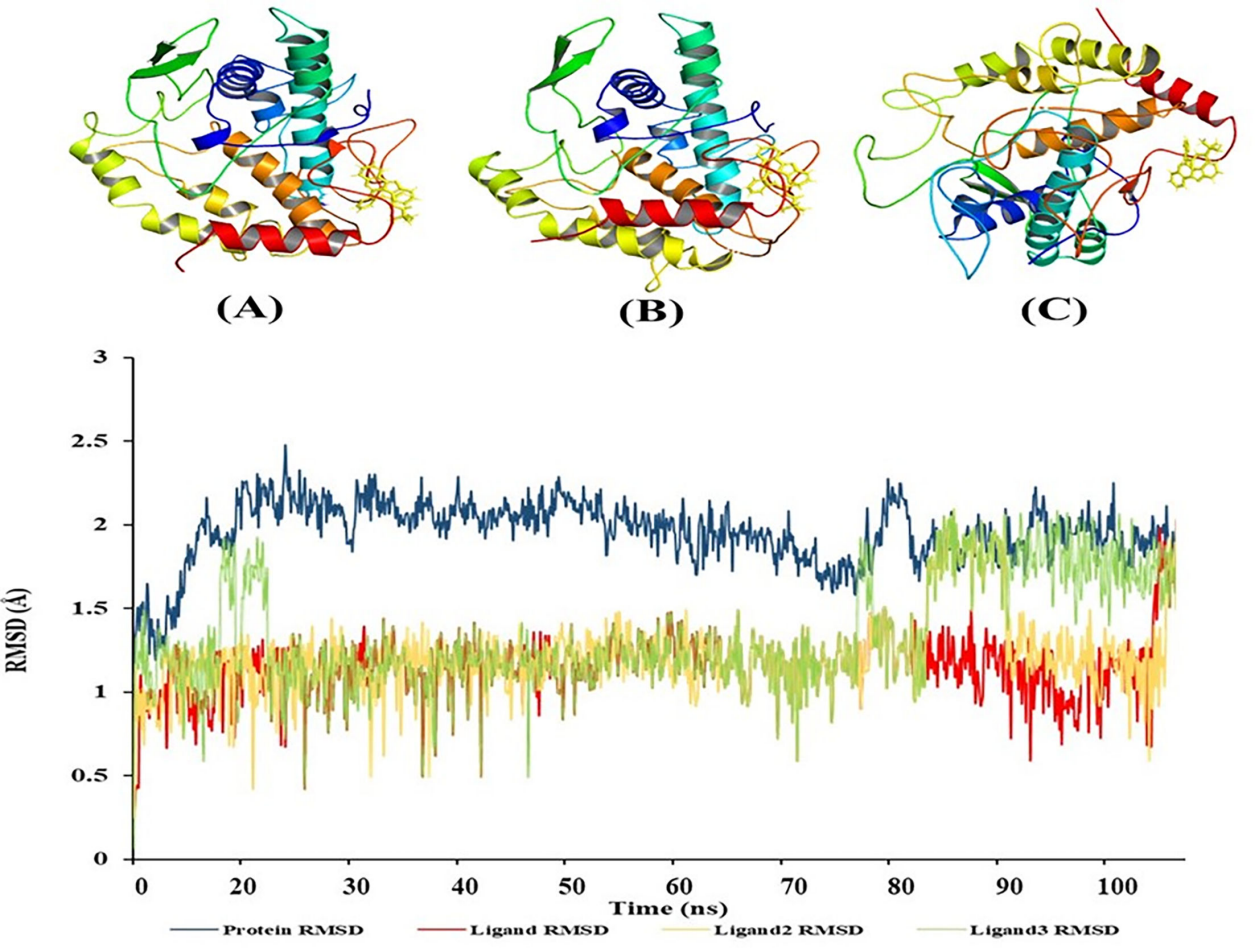
Molecular dynamics simulation diagram showing RMSD values of protein complex and three replicas, namely (A) Ligand RMSD, (B) Ligand 2 RMSD, (C) Ligand 3 RMSD.

The results indicate that conclusions derived from individual simulations are frequently nonreproducible, although those obtained from several shorter replicas are more dependable than those from a singular extended simulation. The requisite number of replicas is dependent upon the question asked and the desired level of credibility. According to our research, a prudent guideline is to do at least three replicas. We conducted triplicate simulations on the peptide system, each lasting 100 ns, resulting in an average simulation duration of 300 ns. The simulations encompass 300 times the system’s folding duration, indicating that we likely attained comprehensive coverage of the free energy space, thereby sampling nearly all conceivable atomic configurations as given in Figure 18.

### Normal mode analysis

**Figure 19:**
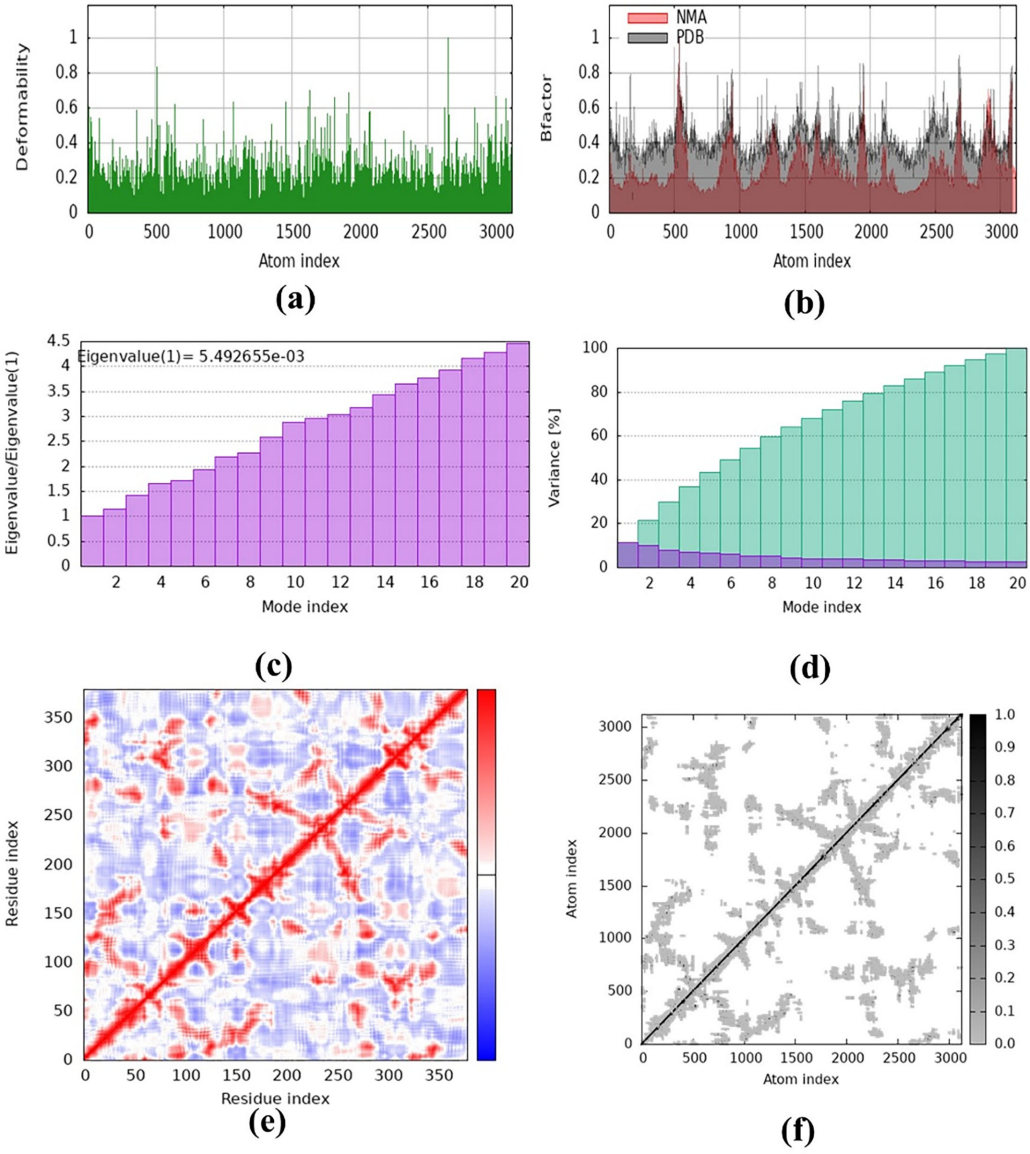
Normal mode analysis of tyrosinase-6g complex. (a) Deformability, (b) B-factor values, (c) Eigenvalue, (d) Variance, (e) Co-variance and (f) elastic network model.

Normal mode analysis (NMA) is a computational technique that analyzes the inherent movements and flexibility of protein–ligand complexes derived from their structural topology. The current study demonstrated by NMA that the tyrosinase–**6g** complex had low eigenvalues, signifying diminished deformability and a stable binding conformation. The structural stability corroborates the experimental observation that **6g** is a potent inhibitor, as robust bioactivity frequently associates with complexes that assume energetically favorable and less deformable conformations. The stability and physical movement of atoms in the docking complex were re-evaluated through molecular dynamics simulation using the iMODS server. Simulations were conducted in NMA, and the results of the tyrosinase-6g docking complex are illustrated in Figure 19. Figure 19a illustrates the deformability graph of both complexes, with hinges highlighting the regions of deformability within the complex. The B-factor (Figure 19b) computes the root mean square value, indicating the uncertainty associated with each atom in the docking complex. The eigenvalues of the tyrosinase-**6g** docking complex were determined to be 5.2946  ×  10–3 (Figure 19c). Figure 19(d) presents the variance matrix graph of residues. The covariance matrix demonstrates (Figure 19e) the coupling between pairs of residues, exhibiting correlated (red), uncorrelated (white), and anticorrelated (blue) motions. The iMODS simulation results indicate that the tyrosinase-**6g** complex exhibits stability (Figure 19f).

### SeeSAR analysis

**Figure 20:**
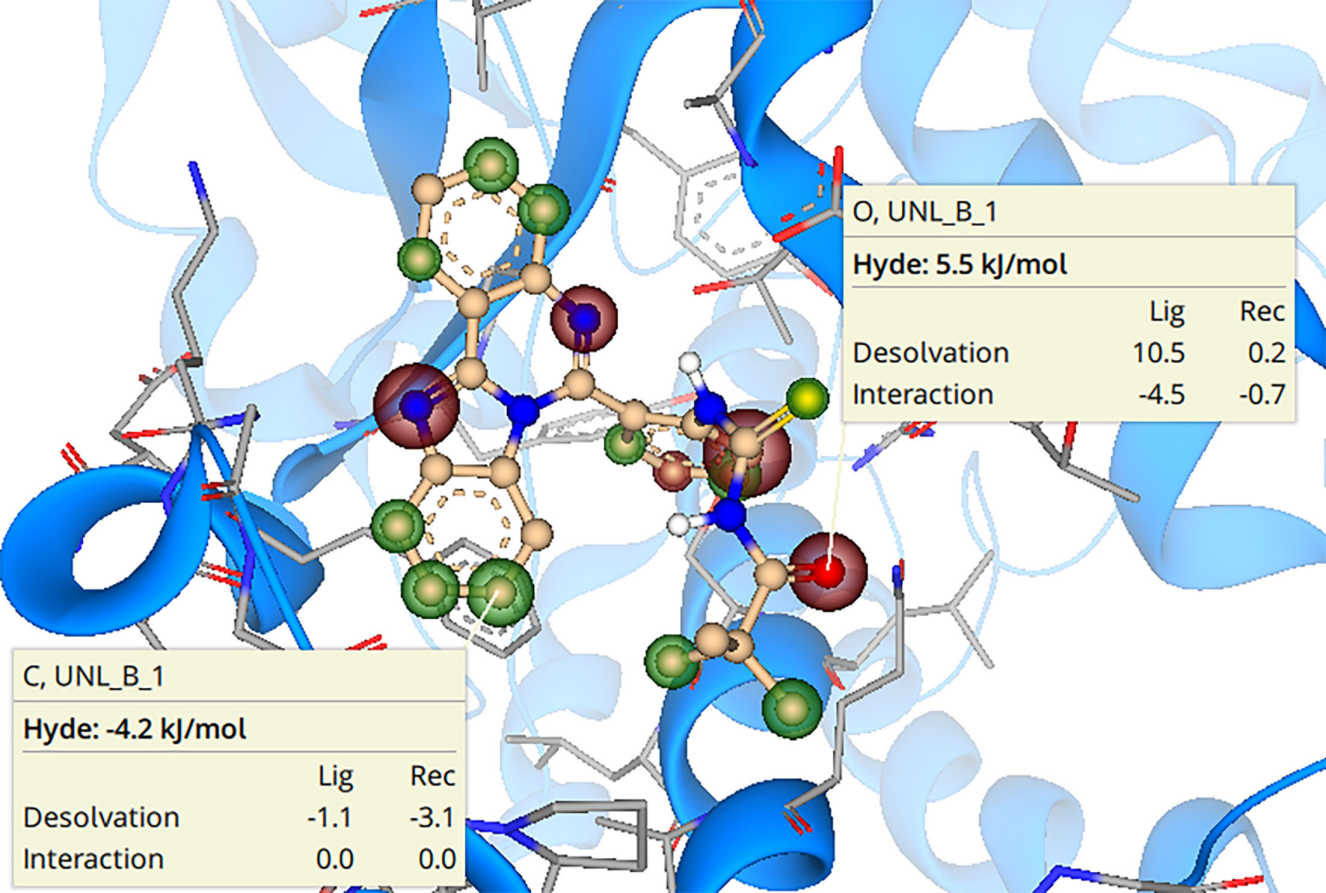
Docked conformations with the corona of the 6g-tyrosinase complex. Red-colored coronas show unfavorable interactions, whereas green coronas represent favorable contributions.

SeeSAR analysis of the best derivative **6g** was executed, which anticipates virtual display of binding interactions. Green-colored coronas were used to positively present the structural components of potent compounds, whereas red-colored coronas were used to favorably convey the structural members that had a detrimental impact on binding interactions. A colorless corona was used to represent structural elements that made little contribution. Corona size determines the contribution of a structural component. SeeSAR visualization of potent derivative **6g** is given in Figure 20. Hyde energy of favorable corona (green colored) for **6g** was –4.2 kJ/mol while the hyde energy of unfavorable corona (red colored) was 5.5 kJ/mol.

### ADMET analysis

The scrutiny of pharmacokinetic properties, i.e. ADME is a prime important aspect for the evaluation of the drug-likeness of lead candidates in successful drug discovery [34]. The ADME parameters of hit molecules were evaluated through the SwissADME Server [35].

Significant gastrointestinal (GI) absorption seen by all compounds suggests their possible therapeutic value. A significant number of the compounds exhibited the inhibition of crucial enzymes, such as CYP2C19, CYP2C9, and CYP3A4 (Table 7.

**Table 7:** ADMET characteristics of synthesized hybrid compounds 6a–h

Molecule	6a	6b	6c	6d	6e	6f	6g	6h
MW	439.53	453.56	467.59	481.61	495.64	509.67	523.69	453.56
Number of H-bond acceptors	3	3	3	3	3	3	3	3
No of H-bond donors	2	2	2	2	2	2	2	2
TPSA	103.41	103.41	103.41	103.41	103.41	103.41	103.41	103.41
iLOGP	3.66	3.9	3.95	4.21	4.52	4.69	4.98	3.83
XLOGP3	5.49	6.03	6.57	7.11	7.65	8.19	8.73	6.06
WLOGP	5.13	5.52	5.91	6.3	6.69	7.08	7.47	5.37
MLOGP	4.01	4.22	4.42	4.62	4.81	5	5.19	4.22
GI absorption	High	High	Low	Low	Low	Low	Low	High
BBB permeant	No	No	No	No	No	No	No	No
CYP2C19 inhibitor	Yes	Yes	Yes	Yes	Yes	Yes	Yes	Yes
CYP2C9 inhibitor	Yes	Yes	Yes	Yes	Yes	Yes	No	Yes
CYP1A2 inhibitor	Yes	No	No	No	No	No	No	No
CYP2D6 inhibitor	Yes	Yes	Yes	Yes	Yes	Yes	Yes	No
CYP3A4 inhibitor	Yes	Yes	Yes	Yes	Yes	Yes	Yes	Yes
Synthetic accessibility	3.1	3.19	3.32	3.45	3.56	3.68	3.8	3.21

The radar charts showing detailed ADME properties are given in Supplementary file.

## Materials and methods

### Chemistry

All commercial solvents and materials, unless otherwise noted, were utilized directly without additional purification. Uncorrected melting points were measured in open glass capillaries. ^1^H NMR spectra were recorded on 300 and 400 MHz spectrometers, and ^13^C NMR spectra were recorded on 75 and 100 MHz spectrometers.

#### *N*-((2-(Benzo [4,5]imidazo[1,2 c]quinazolin-6-yl)phenyl)carbamothioyl)butyramide *(6a)*


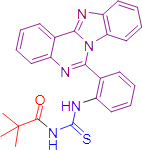


Light brown crystalline solid, mp = 173°C; R*_f_* = 0.41 (hexanes/EtOAc, 7:3), FT-IR (ATR) in cm^-1^; 3187 (N-H), 3030 (Sp^2^ Stretching), 2925, 2860 (H-C, alkyl asym and sym),1698 (C=O), 1580 (C=N), 1525, 1404 (C=C), 1188 (C=S); **^1^H NMR** (300 MHz, DMSO-*d_6_*) δ 12.68 (s, 1H, NH), 11.24 (s, 1H, NH), 8.63 (dd, *J* = 7.8, 1.5 Hz, 1H), 8.34 – 8.25 (m, 1H), 8.01 – 7.70 (m, 6H), 7.59 (td, *J* = 7.6, 1.2 Hz, 1H), 7.47 (ddd, *J* = 8.2, 7.2, 1.1 Hz, 1H), 7.18 (ddd, *J* = 8.4, 7.2, 1.2 Hz, 1H), 6.68 (dt, *J* = 8.5, 1.0 Hz, 1H), 2.21 – 1.98 (m, 2H, CH_2_), 1.24 (h, *J* = 7.3 Hz, 2H, CH_2_), 0.57 (t, *J* = 7.4 Hz, 3H, CH_3_). **^13^C NMR** (75 MHz, DMSO- *d_6_*) δ 180.09 (C=S), 175.7 (C=O), 147.7, 145.5, 144.3, 142.2, 136.8, 132.3, 131.4, 129.9, 129.3, 129.0, 129.0, 128.4, 127.5, 127.4, 125.9, 124.1, 122.9, 119.9, 118.5, 114.5, 37.6, 18.1, 13.3. CHN Elemental analysis: calcd for: C_25_H_21_N_5_OS: C, 66.17; H, 6.14; N, 8.12; S, 9.30/ Found: C, 66.11; H, 5.99; N, 8.01; S, 9.12; HRMS (ESI): m/z Calcd for [C_25_H_21_N_5_OS +H] ^+^ 439.1467, Found 439.1470.

#### *N* -((2-(Benzo[4,5]imidazo[1,2-c]quinazolin-6-yl)phenyl)carbamothioyl)pentanamide (6b)


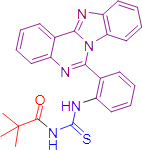


Light brown crystalline solid, mp = 195°C; Rf Rf = 0.48 (hexanes/EtOAc, 7:3) FTIR (ATR) in cm^-1^; 3479 (N-H), 3159 (Sp^2^ Stretching), 3065 (H-C, alkyl asym and sym), 1697 (C = O), 1624 (C = N), 1583, 1436 (C = C), 1160 (C = S);**^1^H NMR** (300 MHz, DMSO-*d_6_*) δ 12.68 (s, 1H, NH), 11.24 (s, 1H, NH), 8.64 (dd, *J* = 7.9, 1.5 Hz, 1H), 8.30 (d, *J* = 8.1 Hz, 1H), 8.01–7.74 (m, 7H), 7.65–7.53 (m, 2H), 7.53–7.42 (m, 2H), 7.18 (ddd, *J* = 8.4, 7.2, 1.2 Hz, 1H), 6.67 (d, *J* = 8.4 Hz, 1H), 2.11 (td, *J* = 7.2, 4.4 Hz, 2H, CH_2_), 1.18 (p, *J* = 7.1 Hz, 2H, CH_2_), 1.02–0.84 (m, 2H, CH_2_), 0.68 (t, *J* = 7.2 Hz, 3H, CH_3_). **^13^C NMR** (75 MHz, DMSO *-d_6_*) δ 179.9 (C = S), 175.9 (C = O), 147.7, 145.5, 144.3, 142.2, 136.7, 132.3, 131.4, 129.9, 129.3, 129.0, 128.4, 127.4, 127.4, 125.9, 124.1, 122.9, 119.9, 118.5, 114.5 (AR-C), 35.5 (CH_2_), 26.7 (CH_2_), 21.6 (CH_2_), 14.0 (CH_3_). CHN Elemental analysis: calcd for: C_26_H_23_N_5_OS: C, 68.85; H, 5.11; N, 15.44; S, 7.07 / Found: C, 68.71; H, 4.97; N, 15.01; S, 6.88; HRMS (ESI): m/z Calcd for [C_26_H_23_N_5_OS + H] ^+^ 453.1623, Found 453.1626.

#### *N* ((2-(Benzo [4,5]imidazo[1,2 c]quinazolin-6-yl)phenyl)carbamothioyl)hexanamide *(6c)*


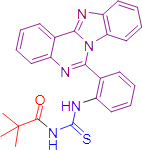


Light yellow crystalline solid, mp = 178°C; R*_f_* = 0.54 (hexanes/EtOAc, 7:3), **FT-IR (ATR**) in cm^-1^; 3294 (N-H), 3151 (Sp^2^ Stretching), 2908, 2849 (H-C, alkyl asym and sym), 1680 (C = O), 1624 (C = N), 1592, 1482 (C = C), 1193 (C = S); **^1^H NMR** (300 MHz, DMSO-*d_6_*) δ 12.48 (s, 1H, NH), 11.34 (s, 1H, NH), 8.53 (dd, *J* = 7.8, 1.5 Hz, 1H), 8.24–8.25 (m, 1H), 8.03–7.69 (m, 6H), 7.53 (td, *J* = 7.6, 1.2 Hz, 1H), 7.42 (ddd, *J* = 8.2, 7.2, 1.1 Hz, 1H), 7.13 (ddd, *J* = 8.3, 7.3, 1.4 Hz, 1H), 6.58 (dt, *J* = 8.4, 1.0 Hz, 1H), 2.48 (t, 2H, *J* = 7.5 Hz, CH_2_), 1.75 (m, 2H, CH_2_), 1.38 (m, 4H, alkyl-H), 0.94 (t, 3H, *J* = 7.2 Hz, CH_3_);**^13^C NMR** (75 MHz, DMSO- *d_6_*) δ 181.09 (C = S), 172.7 (C = O), 143.7, 142.5, 141.3, 142.2, 137.8, 134.3, 132.4, 128.9, 127.3, 127.0, 127.0, 126.4, 125.5, 124.4, 123.9, 123.1, 122.9, 118.9, 117.5, 113.5 (Ar-C), 37.4, 31.5, 24.3, 21.3, 13.9 (alkyl-C); CHN Elemental analysis: calcd for: C_27_H_25_N_5_OS: C, 69.35; H, 5.39; N, 14.98; S, 6.86 Found: C, 69.33; H, 5.37; N, 14.96; S, 6.82 HRMS (ESI): m/z Calcd for [C_27_H_25_N_5_OS + H] ^+^ 467.1780, Found 467.1783.

#### *N*-((2-(Benzo [4,5]imidazo[1,2 c]quinazolin-6-yl)phenyl)carbamothioyl)heptanamide *(6d)* 
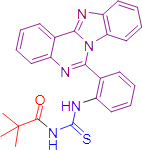


Brown crystalline solid, mp = 242°C; R*_f_* = 0.52 (hexanes/EtOAc, 7:3) **FT-IR (ATR) in cm^-1^;** 3210 (N-H), 3054 (C-H, Ar), 2956, 2867 (H-C, alkyl asym and sym), 1696 (C = O), 1599 (C = N), 1544, 1453 (C = C), 1169 (C = S);**^1^H NMR** (400 MHz, DMSO-*d_6_*) δ 12.58 (s, 1H), 11.33 (s, 1H, NH), 8.62 (d, *J* = 7.8 Hz, 1H, NH), 8.34 (d, *J* = 8.1 Hz, 1H), 8.02–7.65 (m, 6H), 7.49 (t, *J* = 7.5 Hz, 1H), 7.47 (t, *J* = 7.6 Hz, 1H), 7.16 (t, *J* = 7.8 Hz, 1H), 6.57 (d, *J* = 8.4 Hz, 1H), 2.35 (t, 2H, *J* = 7.7 Hz, CH_2_), 1.76 (quin, 2H, *J* = 7.3 Hz, CH_2_), 1.23 (m, 6H, alkyl-H), 0.87 (t, 3H, *J* = 7.4 Hz, CH_3_); **^13^C NMR** (101 MHz, DMSO-*d_6_*) δ 177.5 (C = S), 173.4 (C = O), 145.3, 144.2, 143.7, 141.5, 137.3, 131.8, 131.1, 129.2, 128.6, 128.2, 128.1, 127.7, 127.2, 126.7, 125.8, 123.5, 122.2, 119.6, 118.2, 114.5, 36.5, 30.4, 29.6, 25.1, 21.6, 14.9 (alkyl-C); CHN Elemental analysis: calcd for: C_28_H_27_N_5_OS: C, 69.83; H, 5.65; N, 14.54; S, 6.66 Found: C, 69.80; H, 5.61; N, 14.51; S, 6.61, HRMS (ESI): m/z Calcd for [C_28_H_27_N_5_OS + H] ^+^ 481.1936, Found 481.1939.

#### *N*-((2-(Benzo [4,5]imidazo[1,2 c]quinazolin-6-yl)phenyl)carbamothioyl)octanamide (6e)


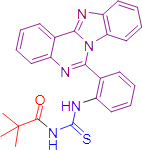


Light brown crystalline solid, mp = 231°C; R*_f_* = 0.51 (hexanes/EtOAc, 7:3) FT-IR (ATR) in cm^-1^; 3258 (N-H), 3050 (C-H, Ar), 2953, 2849 (H-C, alkyl asym and sym), 1693 (C = O), 1697 (C = N), 1538, 1453 (C = C), 1153 (C = S); **^1^H NMR** (400 MHz, DMSO-*d_6_*) δ 12.71 (s, 1H), 11.23 (s, 1H, NH), 8.64 (d, *J* = 7.8 Hz, 1H, NH), 8.31 (d, *J* = 8.2 Hz, 1H), 8.00–7.75 (m, 6H), 7.59 (t, *J* = 7.6 Hz, 1H), 7.48 (t, *J* = 7.7 Hz, 1H), 7.18 (t, *J* = 7.8 Hz, 1H), 6.68 (d, *J* = 8.4 Hz, 1H), 2.11 (tt, *J* = 12.8, 6.3 Hz, 2H, CH_2_), 1.26–1.13 (m, 4H), 1.07 (m, 4H), 0.97–0.89 (m, 2H), 0.83 (t, *J* = 7.2 Hz, 3H, CH_3_). **^13^C NMR** (101 MHz, DMSO-*d_6_*) δ 179.5 (C = S), 175.4 (C = O), 147.3, 145.0, 143.8, 141.7, 136.3, 131.8, 130.9, 129.4, 128.8, 128.5, 128.4, 127.9, 127.0, 126.8, 125.4, 123.6, 122.4, 119.4, 118.0, 114.0, 35.3 (CH_2_), 30.9 (CH_2_), 28.2 (CH_2_), 27.9 (CH_2_), 24.1 (CH_2_), 21.9 (CH_2_), 13.8 (CH_3_). CHN Elemental analysis: calcd for: C_29_H_29_N_5_OS: C, 70.28; H, 5.90; N, 14.13; S, 6.47 / Found: C, 70.12; H, 5.81; N, 14.01; S, 6.12; HRMS (ESI): m/z Calcd for [C_28_H_27_N_5_OS + H] ^+^ 481.1936, Found 481.1939.

#### *N* -((2-(Benzo[4,5]imidazo[1,2-c]quinazolin-6-yl)phenyl)carbamothioyl)nonanamide (6f)


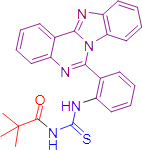


Light brown solid, mp = 163°C; Rf Rf = 0.39 (hexanes/EtOAc, 7:3) **FT-IR (ATR**) in cm^-1^; 3253 (N-H), 3053 (C-H, Ar), 2919, 2848 (H-C, alkyl asym and sym), 1696 (C = O), 1598 (C = N), 1549, 1416 (C = C), 1269 (C = S);**^1^H NMR** (400 MHz, DMSO-*d_6_*) δ 12.64 (s, 1H, NH), 11.31 (s, 1H, NH), 8.65 (d, *J* = 7.9 Hz, 1H), 8.36 (d, *J* = 8.2 Hz, 1H), 8.01–7.69 (m, 8H), 7.58 (t, *J* = 7.5 Hz, 2H), 7.48 (t, *J* = 7.7 Hz, 1H), 7.37 (d, *J* = 7.0 Hz, 1H), 7.19 (t, *J* = 7.8 Hz, 1H), 6.66 (d, *J* = 8.4 Hz, 1H), 1.85 (s, 3H, CH_3_). CHN Elemental analysis: calcd for: C_30_H_31_N_5_OS: C, 70.70; H, 6.13; N, 13.74; S, 6.29 Found: C, 70.72; H, 6.11; N, 13.70; S, 6.27, HRMS (ESI): m/z Calcd for [C_30_H_31_N_5_OS + H] ^+^ 509.2249, Found 509.2252.

#### *N* -((2-(Benzo[4,5]imidazo[1,2-c]quinazolin-6-yl)phenyl)carbamothioyl)decanamide 6g


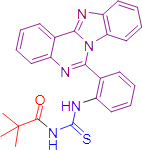


Light brown crystalline solid, mp = 241°C; Rf Rf = 0.55 (hexanes/EtOAc, 7:3) FT-IR (ATR) in cm^-1^; 3231 (N-H), 3044 (C-H, Ar), 1660 (C = O), 1537 (C = N), 1502, 1451 (C = C), 1157 (C = S); **^1^H NMR** (400 MHz, DMSO-*d_6_*) δ 12.55 (s, 1H), 11.27 (s, 1H, NH), 8.61 (d, *J* = 7.7 Hz, 1H, NH), 8.37 (d, *J* = 8.1 Hz, 1H), 8.02–7.72 (m, 6H), 7.57 (t, *J* = 7.5 Hz, 1H), 7.46 (t, *J* = 7.7 Hz, 1H), 7.24 (t, *J* = 7.7 Hz, 1H), 6.64(d, *J* = 8.3 Hz, 1H), 2.45 (t, 2H, *J* = 7.5 Hz, CH_2_), 1.76 (quin, 2H, *J* = 7.2 Hz, CH_2_), 1.31 (m, 12H, alkyl-H), 0.88 (t, 3H, *J* = 6.9 Hz, CH_3_); **^13^C NMR** (101 MHz, DMSO-*d_6_*) δ 178.5 (C = S), 174.4 (C = O), 146.3, 144.0, 143.5, 141.3, 137.1, 133.7, 132.7, 130.4, 129.8, 128.8, 128.5, 127.7, 127.5, 126.5, 125.7, 124.6, 123.4, 119.6, 118.1, 113.0 (Ar-C), 37.2, 31.7, 29.8, 29.5, 29.3, 29.1, 24.4, 23.6, 15.1 (alkyl-C); CHN Elemental analysis: calcd for: C_31_H_33_N_5_OS: C, 71.10; H, 6.35; N, 13.37; S, 6.12 Found: C, 71.12; H, 6.33; N, 13.32; S, 6.15 HRMS (ESI): m/z Calcd for [C_31_H_33_N_5_OS + H] ^+^ 523.2406, Found 523.2409.

#### *N*-((2-(Benzo [4,5]imidazo[1,2 c]quinazolin-6-yl)phenyl)carbamothioyl)pivalamide *(6h)*


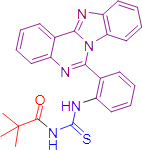


Light brown crystalline solid, mp = 169°C; R*_f_* = 0.43 (hexanes/EtOAc, 7:3) FT-IR (ATR) in cm^-1^; 3178 (N-H), 3056 (C-H, Ar), 1584 (C = O), 1542 (C = N), 1470, 1451 (C = C), 1141 (C = S); **^1^H NMR** (400 MHz, DMSO-*d_6_*) δ 13.29 (s, 1H, NH), 9.48 (s, 1H, NH), 9.19–9.12 (m, 1H), 8.91–8.84 (m, 1H), 8.48 (ddd, *J* = 8.1, 1.3, 0.6 Hz, 1H), 8.41–8.20 (m, 6H), 8.13–8.04 (m, 1H), 7.94 (ddd, *J* = 8.2, 7.2, 1.1 Hz, 1H), 7.63 (ddd, *J* = 8.4, 7.2, 1.1 Hz, 1H), 7.35–7.28 (m, 1H), 1.49 (d, *J* = 1.4 Hz, 9H, *t-butyl*). CHN Elemental analysis: calcd for: C_26_H_23_N_5_OS: C, 68.85; H, 5.11; N, 15.14; S, 7.70 Found: C, 68.45; H, 5.78; N, 15.01; S, 7.30; HRMS (ESI): m/z Calcd for [C_26_H_23_N_5_OS + H] ^+^ 453.1623, Found 453.1626.

### X-ray crystallography

Compounds **6a** and **6b** were examined using X-ray crystallography utilizing a STOE IPDS II two-circle diffractometer with Mo Kα radiation (λ = 0.71073 Å). The data processed using the multi-scan absorption correction X-AREA were analyzed using the SHELX software packages XP in SHELXTL-Plus [36] and SHELXL2016/6 [37] for structure determination and refinement, while ORTEP-3 [38] and PLATON [39] were employed for graphical representations, and results are given in Table 8. The hydrogens of NH and H_2_O were identified in a difference Fourier map and refined isotropically.

**Table 8:** Experimental values for compounds 6a and 6b

Crystal data	6a	6b
Chemical formula	C_25_H_21_N_5_OS·H_2_O	C_26_H_23_N_5_OS
Space group , Crystal system	*P*2_1_/*c,* Monoclinic	*P -*1, Triclinic
*M*_r_	457.54	453.55
Temperature (K)	173	173
*A*, *b*, *c* (Å)	13.8163(8), 7.7180 (2), 21.2068 (11)	10.2422 (6), 15.6054 (8), 15.8013 (9)
β (°)	90.272 (4)	69.909 (4), 87.771 (4), 73.315 (4)
*V* (Å^3^)	2261.34 (19)	2267.0 (2)
*Z*	4	4
*Density (calculated*)	Mg/m^3^	Mg/m^3^
Radiation type	Mo *K*α	Mo *K*α
µ (mm^−1^)	0.18	0.17
Crystal size (mm^3^)	0.21 × 0.11 × 0.08	0.23 × 0.17 × 0.09

**Data collection**		
Diffractometer	STOE *IPDS* II two-circle diffractometer	STOE *IPDS* II two-circle diffractometer
*T*_min_, *T*_max_	0.235, 1.000	0.432, 1.000
Absorption correction	Multi-scan *X-AREA*	Multi-scan *X-AREA*
*R*_int_	0.049	0.039
(sin θ/λ)_max_ (Å^−1^)	0.610	0.610
No. of measured, independent, and observed [*I* > 2σ(*I*)] reflections	26433, 4245, 3498	32312, 8510, 6596

**Refinement**		
Final R indices [I > 2sigma(I)]	R1 = 0.0570, wR2 = 0.1141	R1 = 0.0496, wR2 = 0.1239
R indices (all data)	R1 = 0.0730, wR2 = 0.1205	R1 = 0.0625, wR2 = 0.1311
H-atom treatment	H atoms treated by a mixture of independent and constrained refinement	H atoms treated by a mixture of independent and constrained refinement
Goodness-of-fit on F2	1.116	1.045
Total parameters	314	630
Total reflections	4245	8510
Δρ_max_, Δρ_min_ (e Å^−3^)	0.21,–0.34	0.32,–0.26

Regarding **6a**, the positions of the C-bound hydrogen atoms were determined geometrically at distances of 0.95 Å for aromatic CH, 0.99 Å for CH_2_, and 0.98 Å for CH_3_. These positions were refined using a riding model, applying the constraints of U_iso_(H) = k X Ueq(C), with k values of 1.2 for CH and CH_2_ hydrogens and 1.5 for CH_3_ hydrogens.

Whereas, in the case of **6b**, the C-bound hydrogen atom positions were calculated geometrically at distances of 0.95 Å (for aromatic CH), 0.99 Å (for CH_2_), and 0.98 Å (for CH_3_) and refined using a riding model by applying the constraints of U_iso_(H) = k X U_eq_ (C), where k = 1.2 for CH and CH_2_ hydrogens and k = 1.5 for CH_3_ hydrogens. Atoms [H17C, H17D, C18A, H18C, H18D, H20D, H20E] and [H17E, H17F, C18’, H18E, H18F, C19’, H19E, H19F, H20G, H20H] were disordered over two positions, and they were refined with the occupancy ratios of 0.849(8)/0.151(8). Crystallographic data for the structure reported herein have been deposited with the Cambridge Crystallographic Data Centre as Supporting Information, CCDC No. 2,078,504 for **6a** and CCDC No. 2,078,505 for **6b**. Copies of the data can be obtained through application to CCDC, 12 Union Road, Cambridge CB2 1EZ, UK. (fax: + 44 1223 336033 or e-mail: deposit@ccdc.cam.ac.uk or at http://www.ccdc.cam.ac.uk.

### Hirshfeld surface analysis

The HS analysis is used to visualize how the molecules in the crystal interact with each other. The HS is plotted over d_norm_ and electrostatic potential, and the colors on the surface indicate different types of contacts between atoms, including van der Waals contacts, hydrogen bonds, and nonbonded contacts. The HS shape index is used to analyze stacking interactions between molecules. In the current study, Crystal Explorer 17.5 was used to perform a Hirshfeld surface (HS) study [40] to determine how the molecules in the title compound crystal interact with one another.

### Density function theory (DFT)

Density Function Theory is a molecular computational technique, which is to predict the chemical and physical properties, as well as anticancer potential of compounds. In the current series, the electronic structure of molecules and atoms present in the given compounds was determined using the density functional theory (DFT) calculations under Gaussian09 program [41]. These predictions utilized the B3LYP functional in the 631 G basis set, which is known for its simplicity and efficiency in this context. The present research aims to determine the following details: frontier molecular orbital parameters (FMO), suitable parameters, global and local reactivity descriptors. The result files in FCHK format were examined using Gauss View 6 software [42].

### Molecular docking studies

Molecular docking is a significant method to detect compound binding sites and energy and was performed using Auto Dock Vina software [32]. The RCSB Protein Data Bank was utilized for the protein target tyrosinase, carbonic anhydrase, and alkaline phosphatase using the PDB IDs 4OUA [43], 6FE2 [44], 1ALK [45] respectively. The 2D structures of compounds were drawn with the help of Chemdraw 12.0 [46] and further examined by Pymol [33].

### Molecular dynamics simulation

To generate the dynamic behavior of docked conformations in physiological conditions, molecular dynamics simulations were carried out using Desmond, a program created by Schrodinger LLC [47]. Utilizing the protein preparation wizard, a software program created by Maestro, the protein-ligand complex that was produced by molecular docking was first preprocessed. The generated compound was then further refined using the default parameters and optimized at a pH of 7.0 as given in [48].

An orthorhombic box measuring 10 angstroms was constructed around the protein-ligand complex, and the system was resolved utilizing the TIP3P water model. Additionally, the system was neutralized by incorporating counter ions at a concentration of 0.15 M. Upon completion of the preparation phase, the system underwent equilibration using the NPT and NVT ensembles, which incorporated the Martyna-Tobias-Klein thermostat and Nosé-Hoover barostat. Equilibration was conducted for 20,000 iterations at a temperature of 300 K. Subsequently, the production run was executed for 100 nanoseconds under periodic boundary conditions. The simulated complexes were analyzed through various methods, including RMSD, RMSF, contact map, 2D interaction, and ligand properties [49].

### Normal mode analysis (NMA)

Normal mode analysis (NMA) in internal (dihedral) co-ordinates naturally reproduces the collective functional motions of biological macromolecules. iMODS [50] facilitates the exploration of such modes and generates feasible transition pathways between two homologous structures, even with large macromolecules. This software provides a suitable correlation for the enlarged normal mode analysis approach inside its co-ordinates. The iMODs site provides several parameters, including protein structural deformability, B-factor values, eigenvalue calculations, variance, covariance maps, and elastic network models. The PDB file of the docked complex was submitted to an internet server as an input file, maintaining all default parameters, and the results were obtained within a few minutes, in accordance with the previously specified parameters [51].

### SeeSAR analysis

Visualization of the 3D crystal structures of tyrosinase enzyme was performed with the SeeSAR software [52]. SeeSAR enables quick and interactive assessments of the free energy of binding and torsions [53]. The structure of the tyrosinase enzyme in complex with **6g** was used for visualization of the binding modes for the inhibitor. The HYDE scoring function as embedded in SeeSAR considers the free energy by computing the difference between the unbound and bound states. H-bonds (approximate enthalpy) and dehydration (‘desolvation’, approximate entropy) effects of all nonhydrogen/heavy atoms (HA) [54]. After the overall HYDE computations that run for very few seconds, SeeSAR visualizes the (HYDE-) estimated free energy of binding (ΔG); spherical ‘coronas’ ranging from dark red (unfavorable) to dark green (favorable for affinity) visualize the contribution of an atom and its environment to the overall free energy of binding; corona sizes correlate with the amount of contribution [55].

### ADMET properties

A thorough and improved experimental understanding of ADMET (absorption, distribution, metabolism, excretion, and toxicity) variables is necessary to identify pertinent compounds that show drug-likeness for ongoing in silico studies, according to the methods discussed earlier [56]. The SwissADME online server was utilized to calculate these variables [34].

## Biological evaluation

### Anti-tyrosinase inhibition assay

#### Mushroom tyrosinase inhibition assay

The inhibition of mushroom tyrosinase (Sigma Chemical, U.S.A.) was performed in accordance with previously documented methodologies [57–59]. Each well of a 96-well microplate received 20 µl of mushroom tyrosinase (30 U/ml), 20 µl of the inhibitor solution, and 140 µl of phosphate buffer (20 mM, pH 6.8). After a 10 min preincubation at room temperature, 20 µl of L-DOPA (3,4-dihydroxyphenylalanine, Sigma Chemical, U.S.A.) at a concentration of 0.85 mM was added, and the test plate was incubated for an additional 20 min at 25°C. The absorbance of dopachrome was later quantified at 475 nm using a microplate reader (SpectraMax ABS, U.S.A.). Kojic acid served as a reference inhibitor, whereas phosphate buffer acted as a negative control. The degree of inhibition by the compounds was evaluated as the percentage of concentration necessary to achieve 50% inhibition (IC50). Every experiment was performed three times. IC_50_ values were calculated by nonlinear regression using GraphPad Prism 5.0 (GraphPad, San Diego, CA U.S.A.).

The % Inhibition of tyrosinase was calculated as following:

Tyrosinase % Inhibition= $\left[(Blank-Sample)/B\right]\times 100$ = (*Equation 1*).

#### Alkaline phosphatase inhibition assay

Spectrophotometric analysis, as previously described in publications [58,59], can be used to evaluate the activity of calf intestinal alkaline phosphatase (CIAP). The reaction mixture, including 10 µl of the reagent and 50 mM Tris-HCl buffer (5 mM MgCl2, 0.1 mM ZnCl2, pH 9.5), is preincubated for 10 min following the addition of 5 µl of CIAP (0.025 U/ml). The test mixture was then incubated at 37°C for another half hour after 10 µl of a substrate (0.5 mM p-NPP, para nitrophenyl phosphate disodium salt) was added to initiate the reaction. The alteration in absorbance of released p-nitrophenolate was measured using a 96-well microplate reader (Thermo Scientific Multiskan GO, U.S.A.) at 405 nm. All tests were repeated three times. KH_2_PO_4_ (potassium dihydrogen phosphate) was used as the reference inhibitor for CIAP. The inhibition activity was calculated according to the following equation:


$$Inhibition(\text{\%})=\left[(Blank-Sample)/Blank\right]\times 100$$

IC50 values were designed using 6–8 serial dilutions, and the data were analyzed by nonlinear regression using GraphPad Prism 5 (GraphPad, San Diego, CA, U.S.A.).

#### Carbonic anhydrase inhibition assay

The inhibition of carbonic anhydrase was assessed as previously outlined, with certain modifications [60–62]. The method employed involves the hydrolysis of p-nitrophenyl acetate by carbonic anhydrase, resulting in the formation of yellow p-nitrophenol, which was subsequently analyzed through spectrophotometry. The reaction mixture included 20 µl of inhibitor, 120 µl of 50 mM Tris-Sulfate buffer (pH 7.6 with 0.1 mM ZnCl2), and 20 µl of bovine enzyme (50 U) per well. The components were mixed and preincubated at 25°C for 10 min. A 6 mM stock solution of the substrate p-nitrophenyl acetate was prepared with less than 5% acetonitrile in buffer and utilized fresh for each experiment, with 40 µl added per well to attain a concentration of 0.6 mM per well. The total reaction volume was adjusted to 200 µl. Following a 30 min incubation at 25°C, the components were mixed, and the absorbance of the reaction mixture was measured at 348 nm with a microplate reader (Thermo Scientific Multiskan GO, U.S.A.). Acetazolamide served as the reference standard. Each concentration underwent three separate evaluations. IC50 values were determined through nonlinear regression analysis utilizing GraphPad Prism 5.0 (GraphPad, San Diego, CA, U.S.A.).


$$Inhibition(\text{\%})=\left[(B-S)/B\right]\times 100$$

Here, the B and S are the absorbances for the blank and samples.

## Conclusion

This study successfully identified a series of novel isocryptolepine ‘aza’ type acyl thiourea analogs (**6a**–**6h**) as potent tyrosinase inhibitors. Structural modifications through varying alkyl chain incorporation significantly influenced their hydrophobicity and biological activity. The synthesized compounds exhibited superior tyrosinase inhibitory activity compared with the standard kojic acid, with IC50 values ranging from 0.832 ± 0.03 to 7.945 ± 0.63 μM. Among these, compound **6g** demonstrated the highest potency (IC_50_ = 0.832 ± 0.03 μM) given in Figure 20 and inhibited tyrosinase through a competitive mechanism, making it a promising lead candidate for managing tyrosinase-mediated hyperpigmentation. Beyond tyrosinase, the compounds also showed notable inhibitory effects on other enzymes involved in cancer progression, underscoring their broader therapeutic potential. Molecular modeling studies revealed strong binding interactions with the tyrosinase enzyme, and structure-activity relationship analyses provided valuable insights into the factors driving their inhibitory activity. Additionally, geometry optimization and drug-likeness evaluations confirmed the favorable properties of these analogs as potential drug candidates. These findings highlight the significance of compound **6g** as a multifunctional enzyme inhibitor and pave the way for its further development in the treatment of hyperpigmentation and enzyme-related disorders, including cancer.

## Data Availability

The author confirms that the data supporting the findings of this study are available within the article and its supplementary material.

## Abbreviations

CA: carbonic anhydrase
DFT: density functional theory
SAR: structure–activity relationship

## References

[1] Rao A.R. Sindhuja H.N. Dharmesh S.M. Sankar K.U. Sarada R. Ravishankar GA 2013 Effective inhibition of skin cancer, tyrosinase, and antioxidative properties by astaxanthin and astaxanthin esters from the green alga Haematococcus pluvialis J. Agric. Food Chem. 61 3842 3851 10.1021/jf304609j 23473626

[2] D’Arino A. Caputo S. Eibenschutz L. Piemonte P. Buccini P. Frascione P. et al. 2023 Skin Cancer Microenvironment: What We Can Learn from Skin Aging? Int. J. Mol. Sci. 24 14043 10.3390/ijms241814043 37762344 PMC10531546

[3] Karampinis E. Aloizou A.-M. Zafiriou E. Bargiota A. Skaperda Z. Kouretas D. et al. 2023 Non-melanoma skin cancer and vitamin d: the “lost sunlight” paradox and the oxidative stress explanation Antioxidants (Basel). 12 1107 10.3390/antiox12051107 37237973 PMC10215909

[4] Sample A. He Y.Y 2018 Mechanisms and prevention of UV-induced melanoma Photodermatol. Photoimmunol. Photomed. 34 13 24 10.1111/phpp.12329 28703311 PMC5760354

[5] Boateng S.T. Roy T. Torrey K. Owunna U. Banang-Mbeumi S. Basnet D. et al. 2023 Synthesis, *in silico* modelling, and *in vitro* biological evaluation of substituted pyrazole derivatives as potential anti-skin cancer, anti-tyrosinase, and antioxidant agents J. Enzyme Inhib. Med. Chem. 38 2205042 10.1080/14756366.2023.2205042 37184042 PMC10187093

[6] Yu Z.-Y. Xu K. Wang X. Wen Y.-T. Wang L.-J. Huang D.-Q. et al. 2022 Punicalagin as a novel tyrosinase and melanin inhibitor: Inhibitory activity and mechanism LWT 161 113318 10.1016/j.lwt.2022.113318

[7] Pretzler M. Rompel A 2024 Tyrosinases: a family of copper-containing metalloenzymes ChemTexts 10 12 10.1007/s40828-024-00195-y 39624788 PMC11608171

[8] Yan Z.-F. Guo J. Tian F.-H. Mao X.-X. Li Y. Li C.-T 2015 Active compounds from Schisandra chinensis exhibiting tyrosinase activity and melanin content inhibition in B16 melanoma cells Biotechnol. Bioprocess Eng. 20 814 823 10.1007/s12257-014-0867-x

[9] Lee C.Y. Chen Y.C. Huang Y.H. Lien Y. Huang C.Y 2024 Cytotoxicity and Multi-Enzyme Inhibition of *Nepenthes miranda* Stem Extract on H838 Human Non-Small Cell Lung Cancer Cells and RPA32, Elastase, Tyrosinase, and Hyaluronidase Proteins Plants (Basel). 13 797 10.3390/plants13060797 38592804 PMC10974603

[10] Batool Z. Ullah S. Khan A. Siddique F. Nadeem S 2024 Alshammari A et.al Design, synthesis, and in vitro and in silico study of 1-benzyl-indole hybrid thiosemicarbazones as competitive tyrosinase inhibitors RSC Adv. 14 28524 28542 10.1039/D4RA05015K 39247501 PMC11378339

[11] Pastorekova S. Zatovicova M. Pastorek J 2008 Cancer-associated carbonic anhydrases and their inhibition Curr. Pharm. Des. 14 685 698 10.2174/138161208783877893 18336315

[12] Mboge M.Y. Mahon B.P. McKenna R. Frost SC 2018 Carbonic anhydrases: role in pH Control and Cancer Metabolites 8 19 10.3390/metabo8010019 29495652 PMC5876008

[13] Hussain Z. Mahmood A. Shah Q. Imran A. Mughal E.U. Khan W. et al. 2022 Synthesis and Evaluation of Amide and Thiourea Derivatives as Carbonic Anhydrase (CA) Inhibitors ACS Omega 7 47251 47264 10.1021/acsomega.2c06513 36570246 PMC9773353

[14] Yoshiura K. Nakaoka T. Nishishita T. Sato K. Yamamoto A. Shimada S. et al. 2005 Carbonic anhydrase II is a tumor vessel endothelium-associated antigen targeted by dendritic cell therapy Clin. Cancer Res. 11 8201 8207 10.1158/1078-0432.CCR-05-0816 16299253

[15] Huang H. Huang F. Liang X. Fu Y. Cheng Z. Huang Y. et al. 2022 Afatinib Reverses EMT via Inhibiting CD44-Stat3 Axis to Promote Radiosensitivity in Nasopharyngeal Carcinoma Pharmaceuticals (Basel). 16 37 10.3390/ph16010037 36678534 PMC9864417

[16] Makris K. Mousa C. Cavalier E 2023 Alkaline Phosphatases: Biochemistry, Functions, and Measurement Calcif. Tissue Int. 112 233 242 10.1007/s00223-022-01048-x 36571614

[17] Fernandez N.J. Kidney B.A 2007 Alkaline phosphatase: beyond the liver Vet. Clin. Pathol. 36 223 233 10.1111/j.1939-165x.2007.tb00216.x 17806069

[18] Sharma K.M. Singh N.A. Patnaik R. Tiwari R.P. Singh N.P. Singh Y.P. et al. 2022 Sharks and rays (chondrichthyes, elasmobranchii) from the miocene sediments of Kutch, Gujarat, India: paleoenvironmental and paleobiogeographic implications Hist. Biol. 34 10 29 10.1080/08912963.2021.1893712

[19] Naeem N. Sadiq A. Othman G.A. Yassin H.M. Mughal EU 2024 Exploring heterocyclic scaffolds in carbonic anhydrase inhibition: a decade of structural and therapeutic insights RSC Adv. 14 35769 35970 10.1039/D4RA06290F 39534850 PMC11555472

[20] Jassas R.S 2023 S-heterocycles as potential alkaline phosphatase inhibitors: a medicinal chemistry overview RSC Adv. 13 16413 16452 10.1039/D3RA01888A 37274413 PMC10233329

[21] Hassani B. Zare F. Emami L. Khoshneviszadeh M. Fazel R. Kave N. et al. 2023 Synthesis of 3-hydroxypyridin-4-one derivatives bearing benzyl hydrazide substitutions towards anti-tyrosinase and free radical scavenging activities RSC Adv. 13 32433 32443 10.1039/D3RA06490E 37942455 PMC10629491

[22] Saeed S. Rashid N. Jones P.G. Ali M. Hussain R 2010 Synthesis, characterization and biological evaluation of some thiourea derivatives bearing benzothiazole moiety as potential antimicrobial and anticancer agents Eur. J. Med. Chem. 45 1323 1331 10.1016/j.ejmech.2009.12.016 20056520

[23] Sajid-ur-Rehman A.S. Saddique G. Channar P.A. Larik F.A. et.al Bioorg Med. Chem. 26 3707 3715 10.1016/j.bmc.2018.06.002 29884581

[24] Ahmed A. Saeed A. Ali O.M. El-Bahy Z.M. Channar P.A. Khurshid A A. et.al. Molecules.2021 26 10.3390/molecules26237150

[25] Spackman M.A. McKinnon J.J. Jayatilaka D 2008 Electrostatic potentials mapped on Hirshfeld surfaces provide direct insight into intermolecular interactions in crystals CrystEngComm 10 377 388 10.1039/D3CP01582C

[26] Naghiyev F.N. Tereshina T.A. Khrustalev V.N. Akkurt M. Rzayev R.M. Akobirshoeva A.A. et al. 2021 Crystal structure and Hirshfeld surface analysis of 6-amino-8-(2,6-dichlorophenyl)-1,3,4,8-tetrahydro-2 *H* -pyrido[1,2- *a* ]pyrimidine-7,9-dicarbonitrile Acta Crystallogr. E. Crystallogr. Commun. 77 516 521 10.1107/S2056989021003583 34026256 PMC8100256

[27] Marques MA Gross EK. Berlin Heidelberg Time-dependent density functional theory. InA Primer in Density Functional Theory 2003 Jun 6 (pp. 144-184). Berlin, Heidelberg: Springer

[28] Sulaiman N.M. Taura L.S. Lawal A. Gidado A.S. Musa A Solvent effects on the structural, electronic, non-linear optical and thermodynamic properties of perylene based on density functional theory Journal of Materials Science Research and Reviews 2019 1 3

[29] Ahmed A. Rehman S.-U. Ejaz S.A. Saeed A. Ujan R. Channar P.A. et al. 2022 Exploring 2-Tetradecanoylimino-3-aryl-4-methyl-1,3-thiazolines Derivatives as Alkaline Phosphatase Inhibitors: Biochemical Evaluation and Computational Analysis Molecules 27 6766 10.3390/molecules27196766 36235300 PMC9572939

[30] Amezcua M. El Khoury L. Mobley DL 2021 SAMPL7 Host-Guest Challenge Overview: assessing the reliability of polarizable and non-polarizable methods for binding free energy calculations J. Comput. Aided Mol. Des. 35 1 35 10.1007/s10822-020-00363-5 33392951 PMC8121194

[31] Grochala W. Szarek P 2023 Lessons from the maximum hardness principle InChemical Reactivity 1 277 312 10.1016/B978-0-32-390259-5.00016-0

[32] Trott O. Olson A.J 2010 AutoDock Vina: improving the speed and accuracy of docking with a new scoring function, efficient optimization, and multithreading J. Comput. Chem. 31 455 461 10.1002/jcc.21334 19499576 PMC3041641

[33] Schrodinger L.L 2015 The PyMOL molecular graphics system Version 1 8

[34] Sharif M.S. Riaz N. Ejaz S.A. Saleem M. Momen A. Bashir B. et al. 2025 Molecular conjugates of N-substituted 2,4-dichlorophenoxymethyl and 1,3,4-oxadiazole acetamides as potent 15-LOX inhibitors supported with cytotoxicity, ADME, molecular docking studies and DFT calculations J. Mol. Struct. 1338 142194 10.1016/j.molstruc.2025.142194

[35] Daina A. Michielin O. Zoete V 2017 SwissADME: a free web tool to evaluate pharmacokinetics, drug-likeness and medicinal chemistry friendliness of small molecules Sci. Rep. 7 42717 10.1038/srep42717 28256516 PMC5335600

[36] Sheldrick GM 2008 A short history of SHELX Acta Crystallogr., A, Found. Crystallogr. 64 112 122 10.1107/S0108767307043930 18156677

[37] Crystallogr S.GM.A 2015 Sect. C. Struct Chem 71 3 10.1107/S2053229614024218

[38] Scheibe B. Ivlev S.I. Karttunen A.J. Kraus F 2020 Synthesis and Characterization of the Tetrafluoridochlorates (III) A [ClF4](A= K, Rb, Cs) Eur. J. Inorg. Chem. 2020 1319 1324 10.1002/ejic.202000106

[39] Spek A.L 2009 Platon/squeeze Acta Crystallogr., Sect. D: Biol. Crystallogr 65 148 155 10.1107/S090744490804362X 19171970 PMC2631630

[40] Hirshfeld F.L 1977 Bonded-atom fragments for describing molecular charge densities Theor. Chim. Acta 44 129 138 10.1007/BF00549096

[41] Spackman M.A. Jayatilaka D 2009 Hirshfeld surface analysis CrystEngComm 11 19 32 10.1039/B818330A

[42] Frisch M.J. Trucks G.W. Schlegel H.B. Scuseria G.E. Robb M.A. Cheeseman J.R. et al 2009 Al. Gaussian 09, Revision B.01. Gaussian Inc Wallingford

[43] Dennington R.D. Keith T.A. GaussView M.JM 2016 16. Semichem Inc Shawnee Mission KS version 13 0

[44] Mauracher S.G. Molitor C. Al-Oweini R. Kortz U. Rompel A 2014 Latent and active abPPO4 mushroom tyrosinase cocrystallized with hexatungstotellurate(VI) in a single crystal Acta Crystallogr. D Biol. Crystallogr. 70 2301 2315 10.1107/S1399004714013777 25195745 PMC4157443

[45] Kazokaitė J. Niemans R. Dudutienė V. Becker H.M. Leitāns J. Zubrienė A. et al. 2018 Novel fluorinated carbonic anhydrase IX inhibitors reduce hypoxia-induced acidification and clonogenic survival of cancer cells Oncotarget 9 26800 26816 26800 10.18632/oncotarget.25508 29928486 PMC6003569

[46] Kim E.E. Wyckoff HW 1991 Reaction mechanism of alkaline phosphatase based on crystal structures J. Mol. Biol. 218 449 464 10.1016/0022-2836(91)90724-K 2010919

[47] Cousins K.R 2011 Computer review of ChemDraw Ultra 12.0 J. Am. Chem. Soc. 133 8388 10.1021/ja204075s 21561109

[48] Knapp B. Frantal S. Cibena M. Schreiner W. Bauer P 2011 Is an intuitive convergence definition of molecular dynamics simulations solely based on the root mean square deviation possible? J. Comput. Biol. 18 997 1005 10.1089/cmb.2010.0237 21702691 PMC3145956

[49] Aziz M. Ejaz S.A. Tamam N. Siddique F. Riaz N. Qais F.A. et al. 2022 Identification of potent inhibitors of NEK7 protein using a comprehensive computational approach Sci. Rep. 12 6404 10.1038/s41598-022-10253-5 35436996 PMC9016071

[50] Barclay P.L. Zhang DZ 2021 Periodic boundary conditions for arbitrary deformations in molecular dynamics simulations J. Comput. Phys. 435 110238 10.1016/j.jcp.2021.110238

[51] López-Blanco J.R. Aliaga J.I. Quintana-Ortí E.S. Chacón P 2014 iMODS: internal coordinates normal mode analysis server Nucleic Acids Res. 42 W271 W276 10.1093/nar/gku339 24771341 PMC4086069

[52] Ejaz S.A. Aziz M. Zafar Z. Akhtar N. Ogaly HA 2023 Revisiting the inhibitory potential of protein kinase inhibitors against NEK7 protein via comprehensive computational investigations Sci. Rep. 13 4304 10.1038/s41598-023-31499-7 36922575 PMC10017757

[53] Sankt Augustin 2024) Germany SeeSAR version 14.0.0; BioSolveIT GmbH www.biosolveit.de/SeeSAR

[54] Bietz S. Urbaczek S. Schulz B. Rarey M 2014 Protoss: a holistic approach to predict tautomers and protonation states in protein-ligand complexes J. Cheminform. 6 1 2 12 10.1186/1758-2946-6-12 24694216 PMC4019353

[55] Abida Ejaz S. Sajjad Bilal M. Aziz M. Wani T.A. Zargar S. Fayyaz A. et al. 2023 Computational Exploration of Fluorocyclopentenyl‐purines and‐pyrimidines Derivatives as Potential Inhibitors of Epidermal Growth Factor Receptor (EGFR) for the Treatment of Breast Cancer Chem. Biodivers. 20 e202301190 10.1002/cbdv.202301190 37963090

[56] Ejaz S.A. Aziz M. Fayyaz A. Wani T.A. Zargar S 2024 Computer-aided approach for the identification of lead molecules as the inhibitors of cholinesterase’s and monoamine oxidases: Novel target for the treatment of Alzheimer disease J. Serb. Chem. Soc. 89 177 194 10.2298/JSC230307050E

[57] Ejaz S.A. Hussain M. Channar P.A. Hussain Z. Aziz M. Bux K. et al. 2025 Synthesis of vanillin-coupled 1, 2, 3-triazoles via click reaction: molecular modeling, structure activity correlation by DFT, ADMET and antibacterial studies Chem. Pap. 79 2895 2910 10.1007/s11696-025-03975-z

[58] Raza H. Kazi M.A. Hassan M. Abbas Q 2020 Exploration of Mechanistic Insights of Acemetacin in Melanogenesis Through Zebrafish Model, Enzyme Kinetics, Molecular Docking and Simulation Approaches Pak. J. Anal. Environ. Chem. 21 115 124 10.21743/pjaec/2020.06.14

[59] Hosseini Nasab N. Raza H. Shim R.S. Hassan M. Kloczkowski A. Kim SJ 2022 Potent Alkaline Phosphatase Inhibitors, Pyrazolo-Oxothiazolidines: Synthesis, Biological Evaluation, Molecular Docking, and Kinetic Studies Int. J. Mol. Sci. 23 13262 10.3390/ijms232113262 36362051 PMC9654533

[60] Abbasi M.A. Nazir M. Aziz-ur-Rehman R Siddiqui S.Z. Raza H. Zafar A. et al. 2021 Synthesis, In Vitro, and In Silico Studies of N-(Substituted-Phenyl)-3-(4-Phenyl-1-Piperazinyl)propanamides as Potent Alkaline Phosphatase Inhibitors Russ. J. Bioorganic Chem. 47 1086 1096 10.1134/S1068162021050186

[61] Vanjare B.D. Choi N.G. Eom Y.S. Raza H. Hassan M. Lee K.H. et al. 2023 Synthesis, carbonic anhydrase inhibition, anticancer activity, and molecular docking studies of 1,3,4-oxadiazole derivatives Mol. Divers. 27 193 208 10.1007/s11030-022-10416-6 35344136

[62] Khan F.M. Abbasi M.A. Siddiqui S.Z. Sadiq Butt A.R. Raza H et al. 2021 Convergent synthesis of carbonic anhydrase inhibiting bi‐heterocyclic benzamides: Structure–activity relationship and mechanistic explorations through enzyme inhibition, kinetics, and computational studies J. Heterocycl. Chem. 58 1089 1103 10.1002/jhet.4240

